# The American Society for Microbiology collaboration with the CDC Laboratory Medicine Best Practices initiative for evidence-based laboratory medicine

**DOI:** 10.1128/cmr.00065-18

**Published:** 2024-09-25

**Authors:** Alice S. Weissfeld, Vickie Baselski, Nancy E. Cornish, Colleen S. Kraft, Mark T. LaRocco, Peggy McNult, Irving Nachamkin, James Scott Parrott, Sandra S. Richter, Matthew Rubinstein, Michael A. Saubolle, Robert L. Sautter, James W. Snyder, Joanna Taliano, Donna M. Wolk

**Affiliations:** 1 Microbiology Specialists Incorporated, Houston, Texas, USA; 2 The ASM 7, The American Society for Microbiology’s Committee on Evidence-based Laboratory Medicine, Washington,DC, USA; 3 University of Tennessee Health Science Center, Memphis, Tennessee, USA; 4 Division of Laboratory Systems, Centers for Disease Control and Prevention, Atlanta, Georgia, USA; 5 Emory University, Atlanta, Georgia, USA; 6 M.T.L. Consulting, Erie, Pennsylvania, USA; 7 American Society for Microbiology, Washington, DC, USA; 8 Perelman School of Medicine University of Pennsylvania, Philadelphia, Pennsylvania, USA; 9 Cleveland Clinic, Cleveland, Ohio, USA; 10 Rutgers University, Newark, New Jersey, USA; 11 USA Banner Good Samaritan Medical Center, Banner Health, Phoenix, Arizona, USA; 12 RL Sautter Consulting, LLC, Lancaster, South Carolina, USA; 13 University of Louisville, Louisville, Kentucky, USA; 14 Centers for Disease Control and Prevention, Library Science Branch, Atlanta, Georgia, USA; 15 Geisinger, Diagnostic Medicine Institute, Danville, Pennsylvania, USA; Rush University Medical Center, Chicago, Illinois, USA; Mayo Clinic, Rochester, Minnesota, USA; University of North Carolina Medical Center, Chapel Hill, North Carolina, USA; Vanderbilt University Medical Center, Nashville, Tennessee, USA; Emory University School of Medicine, Atlanta, Georgia, USA

**Keywords:** systematic review, meta-analysis, outcomes, evidence-based medicine

## Abstract

Clinical medicine has embraced the use of evidence for patient treatment decisions; however, the evaluation strategy for evidence in laboratory medicine practices has lagged. It was not until the end of the 20th century that the Institute of Medicine (IOM), now the National Academy of Medicine, and the Centers for Disease Control and Prevention, Division of Laboratory Systems (CDC DLS), focused on laboratory tests and how testing processes can be designed to benefit patient care. In collaboration with CDC DLS, the American Society for Microbiology (ASM) used an evidence review method developed by the CDC DLS to develop a program for creating laboratory testing guidelines and practices. The CDC DLS method is called the Laboratory Medicine Best Practices (LMBP) initiative and uses the A-6 cycle method. Adaptations made by ASM are called Evidence-based Laboratory Medicine Practice Guidelines (EBLMPG). This review details how the ASM Systematic Review (SR) Processes were developed and executed collaboratively with CDC’s DLS. The review also describes the ASM transition from LMBP to the organization’s current EBLMPG, maintaining a commitment to working with agencies in the U.S. Department of Health and Human Services and other partners to ensure that EBLMPG evidence is readily understood and consistently used.

## INTRODUCTION

This review describes the history of Centers for Disease Control and Prevention, Division of Laboratory Systems (CDC’s LMBP) initiatives and integration with the American Society for Microbiology (ASM), specifically the “ASM 7,” a group of clinical microbiologists with diverse backgrounds and interests but a shared belief in the value of clinical microbiology to patient care. The ASM7 met in February 2011 at ASM headquarters in Washington, D.C., and allied with the CDC’s vision to promote evidence-based laboratory medicine practices in clinical microbiology. The alliance resulted in the publication of two Systematic Review (SR) manuscripts, one on rapid detection of bloodstream infections and one on urine pre-analytics (1, 2). These manuscripts represent the first microbiology-focused questions examined by ASM using the CDC DLS LMBP A-6 cycle SR methods (3). After a few adaptations to the A-6 cycle, ASM published a third manuscript focused on *Clostridioides difficile* diagnostic methods (4). Over time, many microbiologists, stakeholders, biostatisticians, medical librarians, and subject matter experts joined the original “ASM 7” team, providing context to the iterations that followed to create ASMs Evidence-based Laboratory Medicine’s Practice Guidelines for Clinical Microbiology’s EBLMPG. Currently, ASM is updating two previous CDC DLS Systematic Reviews, one on blood culture contamination (5) and one on rapid identification of bloodstream infections (1). This review is based on the history of ASM’s adoption and adaptation of the CDC’s LMBP A-6 cycle method.

## WHAT IS AN ASM EVIDENCE-BASED LABORATORY MEDICINE PRACTICE GUIDELINE?

Evidence-based practice (EBP), like evidence-based medicine (EBM), has historical roots going back centuries, but the modern era of popularity dates only to the mid-1990s (6). Its strength lies in “the conscientious and judicious use of current best evidence derived from clinical care research in managing individual patients” (7). The use of EBP is intended to optimize decision-making through the availability of EBP guidelines derived from rigorous analysis of the evidence from well-designed and properly conducted clinical research. This evidence analysis approach is called a SR process; it informs individual medical and scientific decisions with valid summary findings obtained through a systematic search of the available evidence, followed by critique and synthesis.

Coupled with a clinician’s expertise, proficiency, and judgment, important medical decisions, diagnoses, and therapeutic choices (6–8) are made based on the patient’s needs and EBP. A leader in EBP and EBM, the Agency for Healthcare Research and Quality (AHRQ), states that the evidence-based decision-making process involves the following steps (9):

- Converting information needs into focused questions.
- Efficiently identify the best evidence to answer the question and synthesize the evidence.
- Critically appraising the evidence for validity and clinical usefulness.
- Applying the results in clinical practice.
- Evaluating the performance of the evidence in clinical application.

**Fig 1:**
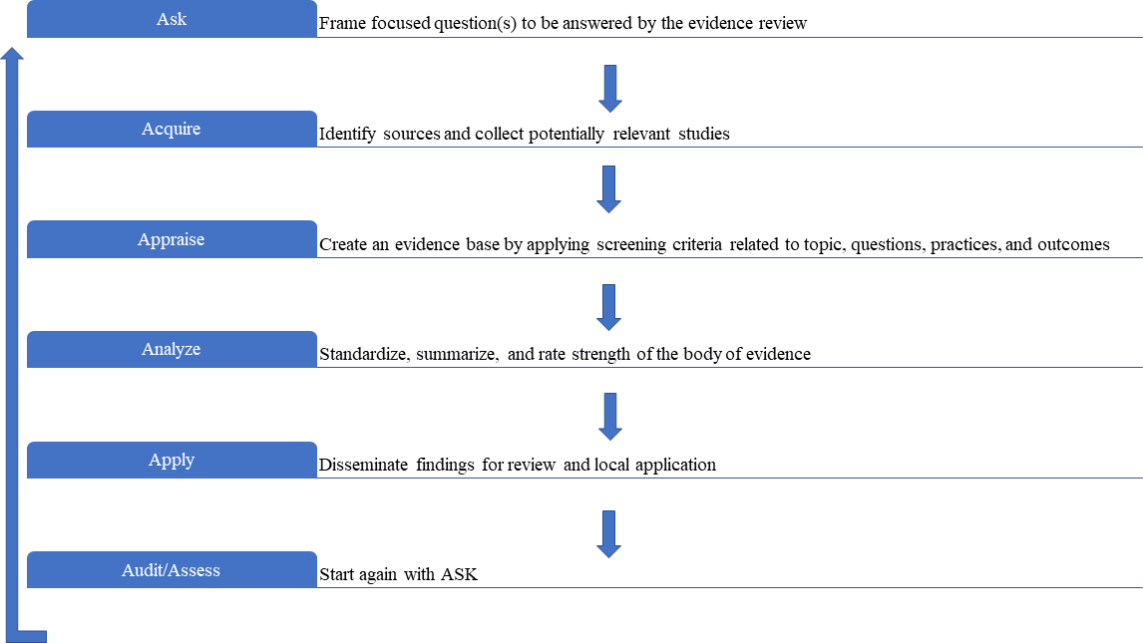
The original LMBP A-6 cycle.

Adding laboratory practices to EBM was a logical step. Importantly, medical decisions include those based on laboratory medicine test results, which comprise approximately 70% of electronic health records (10). In alignment with AHRQ, by 2011, the A-6 method (Fig. 1) was validated by CDC DLS (3) and adopted by ASM. The CDC DLS LMBP A-6 cycle is a method to derive evidence-based laboratory medicine practice guidelines (EBLMPG). EBLMPG emphasizes and utilizes EBP principles and processes to provide best-practice guidelines for clinical laboratories, with linkage to healthcare quality aims and patient outcomes.

In contrast to SR performed in clinical medicine, which focuses primarily on patient-associated outcomes, clinical research in laboratory medicine generally focuses on diagnostic tests’ accuracy, precision, value, and outcomes. Such laboratory evidence often repudiates previously accepted diagnostic tests and replaces them with more robust ones that may better support patient outcomes more efficiently and economically.

### What makes "evidence-based" practice different from consensus-based expert opinion?

When problem-solving is performed by a consensus of expert opinion, the focus is on persons with extensive knowledge and expertise in a given field. Consensus opinion includes the input of several experts in a group whose views are heard and understood. Consensus is neither an absolute agreement between the expert discussion nor the majority’s preference. Instead, the consensus process includes collaborative, cooperative, egalitarian, inclusive, and participatory decision-making; the resulting outcome reflects the best interest of the whole, achievable at the time, and supported by all. As expected, any group of experts has varied experience levels and different perspectives, which influence their input to the overall discussion and their practice recommendations. Proposals are generated collaboratively, unsatisfied concerns are addressed, and the recommendations are modified to maximize general agreement. Through such cooperative decision-making, the discussers consent overall to progress in settling the issue or question at hand, even if there is disagreement on select topics. Thus, the final solution creates a consensus opinion, often defined as an “acceptable solution” in which participants mutually agree on the solution, essentially representing the collective opinion of each discussion group member.

There are many examples of consensus-driven guidelines based on expert opinion and published by scientific organizations in their area of expertise and overlapping topics. Within clinical microbiology, the organizations providing most of the guidelines include the American Society for Microbiology (ASM), the Clinical and Laboratory Standards Institute (CLSI), the Infectious Diseases Society of America (IDSA), and the College of American Pathologists (CAP). Each organization has its own, albeit often similar, approach to publishing consensus guidelines, which are summarized in Table 1. In 2010, ASM aimed to replace the Cumitechs with Practical Guidance for Clinical Microbiology (PGCM) documents, now published in Clinical Microbiology Reviews (CMR), ASM Press. CMR editors prioritize previous Cumitech subjects and new subjects, assembling a group of experts with different expertise to work on the topic being reviewed. The group used evidence-based literature, expertise, and the team’s consensus to develop documents that provide general guidance for clinical microbiologists, emphasizing current diagnostic methods and their appropriate implementation. These PGCM documents link microbiologic practice to current clinical and scientific issues.

**TABLE 1:** Laboratory guideline attribute by organization^*a*^

Attribute and example organizations	CLSI	CAP^*b*^	IDSA	CDC-LMBP^*c*^	ASM-Cumitech and CPG	ASM-EBLMPG
Consensus opinion after routine literature review expert opinion	Yes	Sometimes	Sometimes	No	Yes	No
Consensus based on evidence from a systematic literature review	No	Yes	Yes	Yes	No	Yes
Consensus based on evidence from a systematic literature review with meta-analysis	No	No	No	Yes	No	Yes
Multidisciplinary panels are convened from major stakeholders	Yes	Yes	Yes	Yes	No	Yes
Training provided for the multidisciplinary panelists	No	Unknown	Unknown	Yes	No	Yes
Librarian included	No	Unknown	Unknown	Yes	No	Yes
Methodologist included	No	Yes	No	Yes	No	Yes
Biostatistician included	No	No	Optional	Yes	No	Yes
Patient advocates included	No	Sometimes	Unknown	Yes	No	No
Documented requirements for guideline creation	Yes	Yes	Yes	Yes	Yes	Yes
Uses GRADE criteria for guidelines	No	No	No	Yes	No	Yes
IOM compliance or posted to AHRQ or former NGC (de-funded)	No	No	Yes	Yes	No	Yes
Includes information about worldwide regulatory agencies and compliance	Yes	No	No	No	No	No
May included industry or government representation with COI disclosure	Yes	Optional	Optional	Yes	Yes	Optional
Includes unpublished grey literature or data	No	Yes	No	Yes	Maybe	No
Includes possible harm assessment	No	Yes	No	Yes	No	Yes
Includes bias assessment	No	No	No	Yes	No	No
Includes cost assessment	No	Yes	Optional	Sometimes	No	Sometimes

^*a*^ Clinical Laboratory Standards Institute, CLSI; College of American Pathologists, CAP; Infectious Disease Society of America, IDSA; CDC Laboratory Medicine Best Practices, CDC-LMBP; ASM Practical Guidance for Clinical Microbiology (PGCM) or Cumitech, ASM-CPG; ASM Evidence-based Laboratory Medicine Practice Guidelines, ASM-EBLMPG.

^*b*^ They also consider the benefits/harms, value, and cost of each guideline and include open comment period feedback from stakeholders and experts.

^*c*^ Published to AHRQ.

In contrast to the consensus process, the main focus of EBP is on the critical evaluation and synthesis of evidence derived from empirical research gathered by a SR process. Analysis of SR data is then combined with the expertise of clinicians, scientists, and patient advocates in making the final decisions about the care of individual patients (4). The goal of EBP is to benefit patient outcomes and achieve healthcare quality aims. EBP exposes limitations and research gaps in the existing evidence base while emphasizing the most efficient and effective patient care processes and practices. To align with EPB, LMBP uses the best available, systematically evaluated evidence combined with clinical acumen, scientific expertise, and knowledge of patient-centric healthcare and laboratory operations. All aspects are used to weigh any evidence-based laboratory guideline for its use in patient care with decisions based on SR with metanalysis. An SR need not include a meta-analysis (especially when studies are both limited and highly heterogeneous), and when this is the case, a narrative synthesis without statistical assessment may be more appropriate. However, combining a SR with a meta-analysis (Fig. 3) is optimal (11, 12).

### What is a systematic review with meta-analysis?

A Systematic Review (SR) is a literature review designed to answer a defined research question by collecting, summarizing, and critically analyzing all empirical evidence (e.g., multiple research studies or manuscripts) that fits pre-specified eligibility criteria. The hallmarks of an SR leading to an EBP guideline are 3-fold: first, the literature search is extensive, systematic, and transparent; second, the risk of bias (RoB) is assessed for all included studies; and third, the strength of the synthesized evidence is graded in a standardized fashion. The SR is then followed by a meta-analysis that uses statistical methods to summarize the results of selected studies from the SR (3).

For individual treatment questions, SRs of extensive collections of randomized clinical trials (RCT) are the ideal evidence base but do not necessarily include recommendations. EBP is not restricted to RCTs but can draw on other studies, published or unpublished (e.g., gray literature) that meet pre-established quality criteria and contain relevant information for the project questions(s) (13). Importantly, questions for laboratory practice extend beyond individual treatment questions to include laboratory process improvement questions, where the focus is on diagnostic accuracy questions or the implementation of procedures or interventions within organizations that could improve the health of individual patients. For questions like these, other study designs are more appropriate (14).

### Limitations of systematic reviews

Systematic Reviews, by their very nature, generally seek to answer a narrow and specific question—they typically do not cover a wide breadth of related topics. Also, conducting a meta-analysis does not guarantee that there will be no bias in the individual studies that contribute to the synthesis. Thus, methodological flaws in individual study design may also contribute to bias. One set of authors cautions users of SRs to carefully consider the quality of the product and adhere to the dictum “caveat emptor” (buyer beware) (15).

These limitations and other issues led to the request by the U.S. Congress that a set of standards be created, which could be used to develop trustworthy clinical practice guidelines. The standards encompass issues like transparency, conflicts of interest, guideline development, group composition, external review, strength of recommendations, and guideline updates. At the request of the U. S. Congress, the Institute of Medicine (IOM) developed a set of standards for developing rigorous, trustworthy clinical practice guidelines (https://nap.nationalacademies.org/resource/9728/To-Err-is-Human-1999--report-brief.pdf). The IOM Report that resulted was called “Clinical Practice Guidelines We Can Trust” and was published in 2011 (16). Since that report, different tools have been developed to help both SR authors and readers evaluate the RoB in SRs (13, 17, 18).

## HISTORY OF EVIDENCE-BASED GUIDELINES IN LABORATORY MEDICINE AND CLINICAL MICROBIOLOGY

### The Cochrane Consumer Network

The Cochrane Consumer Network (https://consumers.cochrane.org/) is the original clinical medicine group performing SRs to generate clinical evidence. It is an international network headquartered in the United Kingdom and is home to over 7,500 SRs. The CDC DLS LMBP includes the A-6 cycle method for SRs and was the first evidence-based method dedicated to quality issues in laboratory medicine. The CDC DLS commissioned the development of a report, which was published in May 2008, called Laboratory Medicine: A National Status Report (19). The report’s content was developed based on the input of a committee composed of technical experts and numerous diverse stakeholders involved with laboratory medicine. The report aimed to lay the groundwork for transforming laboratory medicine over the following decade. The LMBP initiative was conceived, in part, to address the evaluation gap identified between laboratory medicine practices and their impact on national healthcare quality aims and patient outcomes. The plan was to develop methods for systematically reviewing and evaluating laboratory quality improvement studies to identify effective laboratory medicine practices linked to national healthcare quality aims and improved patient outcomes. The result was the development of the robust CDC DLS LMBP A-6 SR method, based on validated evidence-based SR methods used in clinical medicine. Since most errors occur in the pre-analytical and post-analytical testing phases, laboratory practices in these areas are the focus of LMBP efforts (19). The inclusion criteria are outlined in the IOM Report (16) and were adopted by the now-defunded National Guideline Clearinghouse (NGC) in 2013 All published LMBP Systematic Reviews, and the first two ASM guidelines were accepted and posted by NGC before it was discontinued.

The American Association for Clinical Chemistry (AACC) was the first clinical laboratory society to publish laboratory practice guidelines. The National Academy of Clinical Biochemistry (NACB) initially published guidelines for point-of-care testing (20), developed like consensus guidelines. Since then, CAP started publishing evidence-based guidelines for laboratory medicine, which qualify for and are placed in the NGC (21). The ASM also started producing evidence-based guidelines that address clinical microbiology laboratory issues. The following sections address ASM’s approach to EBLMPG, which is based on the CDC DLS LMBP A-6 cycle methods.

### The CDC DLS LMBP A-6 cycle method

The CDC DLS LMBP A-6 cycle method is summarized in Table 2 and is compared with SR steps common to all evidence-based guideline approaches. Steps A1-A6 of the CDC DLS LMBP A-6 cycle guide the LMBP SRs. LMBP data collection and analysis are distinct but interrelated processes, followed by a meta-analysis (3). A team of individuals with specific functions conducts the SR. These team members work in a highly coordinated manner to conduct the SR and meta-analysis to develop an EBP guideline.

**TABLE 2:** Steps in performing a systematic review and the relationship to the Laboratory Medicine Best Practice A-6 method

Step	Description	A-6 step
Step 1	Formulate a question for review. Once the question is agreed upon, it should not be changed.	ASK-A-1
Step 2	Identify all relevant studies. American Society for Microbiology (ASM) works with a medical librarian who has been specially trained in performing literature searches for Systematic Reviews. The Centers for Disease Control and Prevention (CDC) librarian is one of the individuals who worked with ASM. The librarian and the rest of the team drill down to keywords or strings of words that should be used to retrieve the available literature.	ACQUIRE-A-2
Step 3	Assess the quality of the studies. There are several ways to do this. Originally, clinical medicine studies were performed using the “Cochrane” method. This method was part of the original British method for Systematic Reviews and uses the QUADAS method for assessing bias and quality	APPRAISE-A-3
Step 4	Summarize the evidence from the total body of evidence. Data analysis consists of using statistical analyses to understand similarities and differences between studies using meta-analysis.	ANALYZE-A-4
Step 5	Interpret the findings. At this point, the Systematic Review (SR) question should be answered based on the findings and conclusions drawn from the total body of evidence.	ANALYZE-A-4
Post SR	Apply the findings to actual clinical practice. Assess the impact of the practice	APPLY-A-5 ASSESS-A-6

The A-6 cycle consists of six steps described in the section, “How are Systematic Reviews Performed Using the CDC DLS LMBP A-6 Cycle Method?” To promote transparency, the A-6 process is open to all relevant stakeholders, including the public (3). The “A’s” definitions (Ask, Acquire, Appraise, Analyze, Apply, and Assess) assure reproducibility and minimize bias while facilitating guideline development and updates. It is an expectation that if an A-6 cycle SR were repeated by a different team using the same method and criteria, they should achieve the same findings. One of the differences in the LMBP A-6 cycle method is the inclusion of unpublished data, recommended by The Cochrane Collaboration to mitigate publication bias (1, 2, 22). Grey data can originate from laboratories conducting Quality Improvement (QI) projects or cost-benefit business data that could be included in the review to benefit patient care.

In 2010, Dr. Robert Kolter, the ASM President, convened 10 clinical microbiology leaders at ASM to review current products and services, brainstorm new ideas, and plan for the future. The intent was to make sure that the clinical microbiology community viewed ASM as responsive to their concerns. One important new initiative was to establish the ASM Professional Practice Committee. The Professional Practice Committee established the EBLMPG Subcommittee to ensure clinical microbiologists addressed laboratory knowledge gaps through an SR process and ultimately improved health outcomes by developing and disseminating evidence-based guidance to laboratorians, patients, clinicians, and other decision-makers. The anticipated guidance would describe laboratory interventions that are most effective for patients under specific circumstances. In addition, it was clear that the Centers for Medicare and Medicaid Services (CMS) was critically evaluating practices that would be reimbursed based on determinations that payment was not appropriate for services for which evidence to support the approach was not available (i.e., not “medically necessary”). Other payers generally follow CMS payment rules, and loss of reimbursement would be detrimental to the clinical laboratory; therefore, evidence to support best practices was deemed imperative.

### Collaboration between CDC DLS and ASM

The CDC DLS had developed and validated the methodology that became the LMBP “A-6 Cycle” method (3) and was interested in piloting the project with external clinical laboratory partners. In 2010, with the collaboration of CDC DLS and ASM, Dr. Peter Gilligan, Dr. Alice S. Weissfeld, and Peggy McNult worked collaboratively to expand the use of the LMBP A-6 cycle in clinical microbiology and invited seven members to participate. There were several reasons ASM decided to adopt and adapt the CDC DLSLMBP A-6 cycle method for Systematic Reviews. First, the LMBP A-6 cycle method focuses on identifying evidence-based practices that support the six healthcare quality aims of the Institute of Medicine (IOM), now renamed the National Academy of Medicine. These aims are to provide healthcare that is patient-centered, safe, timely, effective, efficient, and equitable (23).

CDC DLS trained the ASM EBLMPG committee members on the LMBP A-6 cycle method in 2011. Each of the seven committee members selected a topic, developed a question, and performed a prequalification literature search under the guidance of the CDC DLS team. Topics were presented at a weekend meeting, and the ASM members selected the top three to investigate. All guidelines are freely accessible upon publication, aligning with CDC and NGC recommendations so that all clinical laboratories have access and can utilize the best practice recommendations therein.

ASM shadowed the CDC DLS team as they researched the first topic: "What practices are effective at increasing timeliness for providing targeted therapy for those patients who are admitted for or are found to have bloodstream infections (e.g., positive blood cultures) to improve clinical outcomes (reductions in length of stay, antibiotic costs, morbidity, and mortality)?" (1). In 2013, ASM and CDC DLS signed a Memorandum of Understanding to solidify their relationship. They embarked on developing a second guideline, also chosen by ASM committee members: "Does optimizing the collection, preservation, and transport of urine for microbiological culture improve the diagnosis and management of patients with urinary tract infection?" (2). Testing for C. *difficile* was the third guideline topic: some questions it answered were as follows: “How effective are NAAT testing practices for diagnosing patients suspected of *Clostridioides difficile* infection?” “What is the diagnostic accuracy of Nucleic Acid Amplification Tests (NAAT) versus either toxigenic culture or cell cytotoxicity neutralization assay?” and “What is the diagnostic accuracy of a glutamate dehydrogenase (GDH)-positive EIA followed by NAAT versus either toxigenic culture or Columbia colistin naladixic acid agar (CCNA)?” (4). Pre-surveys for the urine pre-analytic and the C. *difficile* testing guidelines were launched in early 2016 and were the last pre-surveys performed.

Finally, ASM entered into a 5-year cooperative agreement with CDC DLS designed to address issues with the final steps of the LMBP A-6 process (A-5, APPLY, and A-6, ASSESS). The agreement intended to establish metrics to measure EBLMPG’s dissemination and promotion, awareness, familiarity, adoption, and implementation (24, 25). The agreement was designed to help organizations develop lasting and sustainable institutional knowledge with qualitative or quantitative methods using free or relatively inexpensive approaches. The survey instruments for all the activities mentioned above were reviewed and approved by the Office of Management and Budget (OMB Control Number 0920–1096; Expiration Date 01/31/2019). The surveys were intended to be completed by stakeholders pre- and post-publication of guidelines to assess guideline impact on laboratory practice.

### ASM members on the LMBP committee

Three groups are integral to ASM’s use of the LMBP A-6 cycle method: (i) the “Review Team” (aka the Core Team), comprised of up to 10 individuals; (ii) the Expert Panel of five individuals; and (iii) the LMBP Workgroup (15–20 individuals depending on the focused questions selected for the project) (3). All original and revised roles are listed in Table 3. The Review Team is led by the Review Coordinator, who works with the Medical Librarian and assigns articles to the Expert Panel consisting of subject matter experts on the topic being investigated; the panel members perform the data abstractions. The Review Coordinator ultimately develops the manuscript detailing the SR process and its conclusions. The Review Team also includes the Technical Lead, a clinical microbiologist with unique expertise regarding the topic under review. Other members include the CDC Liaison, a member of the LMBP team, and the ASM Liaison, the Chair of the Subcommittee on Evidence-based Medicine for ASM. Finally, the Biostatistician (more specifically, a Meta-biostatistician) serves as a member of the Review Team and participates in all discussions leading up to the actual data abstraction, audits, and consensus of the abstractors. The Meta-biostatistician also coordinates the statistical review of the findings, including the meta-analysis upon which recommendations are made and graded. The LMBP Workgroup, a group of multidisciplinary stakeholders convened by CDC DLS, provides an independent external review of the SR activities to identify conflicts of interest, issues with the SR protocol and execution, and bias (financial, unconscious, and intellectual) that may have been inadvertently introduced.

**TABLE 3:** Team members in the LMBP A-6 process

Title	Roles in CDC/ASM teams	Role in current ASM EBLMPG
Review coordinator	Process oversight	Yes
Technical/Scientific lead	Scientific oversight	Yes
ASM Liaison	ASM/CDC liaison	Yes
CDC Liaison	ASM/CDC liaison	No
LMBP workgroup (CDC)	Independent advisory and review group convened by CDC	Yes^*a*^
Expert Panel	Reviewers for inclusion/exclusion criteria, performing extractions and adding scientific subject-matter expertise	Yes
External Extraction Team	Not applicable	Yes^*b*^
Technical assessment Team	Appraisers of final recommendations	No
Technical review team	Reviewers for final document pre-publication	No
Medical librarian	Support for data procurement and management	Yes
Meta-Biostatistician	Support for data analysis including meta-analysis	Yes
Stakeholders	Reviewers for final document post-publication	No

^*a*^ The role differs with ASM. CDC no longer assigns the roles and the workgroup performs only bias assessment, not the full extraction. The full extraction and scoping reviews for ASM are now performed by the Rutgers External Extraction Team.

^*b*^ A new team added by ASM for initial extraction and scoping reviews.

## ADAPTING LMBP TO ASM’S CURRENT EBLMPG PROCESS

Since 2011, several notable changes occurred when ASM further adapted the EBLMPG process. In 2016, the QUADAS-2 was added to the EPLMPB, at that time for the assessment of diagnostic accuracy for the *C. difficile* guideline (4). QUADAS stands for Quality Assessment of Diagnostic Accuracy Studies, and the tool was originally developed in 2003 specifically for meta-analysis of diagnostic accuracy studies (26). QUADAS and QUADAS-2 were specifically developed to facilitate the comparison of studies with heterogeneous study designs (27). These instruments include questions to assess bias, sources of variation, and reporting quality. QUADAS-2 is recommended by the AHRQ, the Cochrane Collaboration, and the United Kingdom National Institute for Health and Clinical Excellence.

By 2019, CDC DLS MUA and the cooperative agreement ended, and Dr. Colleen Kraft assumed the leadership of the ASM EBLMPG from Dr. Alice S. Weissfeld. In 2021, ASM discontinued collaboration with the CDC DLS and named Rutgers University, Newark, NJ, as a new collaborator under the direction of Dr. J. Scott Parrott, and Dr. Esther Babady became the new EBLMPG leader.

Table 3 provides an overview of ASM’s adaptation of roles from the CDC DLS LMBP SR process from 2010 to 2023. To speed and streamline ASM’s EBLMPG process, in 2019, grey data were excluded from the ASM EBLMPG process based on the limited number of abstract data that met the quality criteria and the requirement for IRB review. Additionally, Rayyan web-based software (28) was used to drive the title and abstract review and document consensus among the experts for inclusion and exclusion. Before, Rayyan, the Core Review team used spreadsheets to document the review process. Finally, in 2019, ASM adopted the use of artificial intelligence (AI)-enhanced title and abstract screening resource (29), the SR Data Repository Plus web-based software (SRDR+), which is now the method of choice for documenting the process of moving the full literature review to the publication list that meets the criteria of the research question before the data abstractions.

In 2021, the process further diverged from the classic LMBP process. Since then, there is no longer a CDC Liaison on the Core Review Team. In addition, the Expert Panel no longer solely performs the discrete data abstractions. Recognizing the differences in content versus statistical expertise, the Expert panel co-extracts data from the included articles along with specially trained students and faculty at Rutgers University into the AHRQ Systematic Review Data Repository (30). The Expert panel also performs bias assessment of all publications and harmonizes results. Finally, the LMBP Workgroup no longer exists as part of the EBLMPG process.

One of the most important issues for any SR is for all participants to use the same extraction template (31). The SRDR electronic platform is maintained by Brown University (Providence, RI) at the behest of AHRQ. In this format, scoping reviews are completed in conjunction with Rutgers faculty and the Core Review Team before the full systematic reviews to determine which research questions are most likely to have sufficient evidence to answer the question. An example scoping review was published by Rubenstein et al., describing rapid diagnostic practices for positive blood cultures (32).

## HOW ARE ASM SYSTEMATIC REVIEWS PERFORMED USING THE CDC DLS LMBP A-6 CYCLE METHOD?

### ASM’s experience with ask step – A-l

The initial LMBP topics were selected by the clinical microbiologists dubbed “ASM 7.” The development of focused questions was guided by the PICO framework (Population, Indicator/intervention/test, Comparator/control, and Outcome). The focused question reflected the problem, including (i) population involved, (ii) practices/intervention used, (iii) intermediate outcomes (e.g., laboratory parameters, impact on workload, and turn-around-times), and (iv) health/healthcare outcomes (e.g., morbidity and mortality). The latter may also include economic impact information. Analytic frameworks were established for these focused questions to depict how implementing selected interventions/practices can lead to targeted quality aims and outcomes.

### ASM’s experience in developing analytic frameworks

Systematic Reviews using the CDC DLS A-6 cycle method begin with the preparation of an analytic framework, a collaborative effort between the SR team, the expert panel, the meta-biostatistician, and others as required for the particular project (librarians, evaluators, etc.). The analytic framework visually outlines the question(s) being asked and identifies the bodies of evidence to be included. Furthermore, analytic frameworks define and clarify the scope of the topic to be investigated by promoting a “structured” methodological approach and promoting transparency, consistency, and compatibility of results in external review. Analytic frameworks are a common method for organizing and linking the separate evidence analysis questions and are a type of logic model (33, 34), whereas an analytic framework may serve as a major outcome of a SR (e.g., by organizing a complex process into a coherent “map” or model) (35). At the very least, an analytic framework provides a crucial planning template for the evidence analysis project that accomplishes the following tasks:

#### Formulate answerable questions

Questions that link “near neighbor” items in the model are often more feasible than questions that try to link early processes to “downstream” outcomes. For example, a question focusing on pre-analytical processes (e.g., appropriate test or test algorithm selection) may connect easily with a question about diagnostic test accuracy. In contrast, a question that seeks to link pre-analytical processes to downstream patient health outcomes may be unanswerable by the current evidence except through inference and consideration of other bodies of evidence (e.g., the availability of proven patient management strategies triggered by test results).

#### Prioritize among questions

When several different review questions are asked about a laboratory medicine process, an analytic framework can help the SR team prioritize questions and focus the search and analysis efforts.

#### Prepare for analysis

The question being asked will determine not just the data to be extracted from the individual studies but will also shape the structure of the data extraction tool and the type of synthesis (e.g., meta-analysis or narrative synthesis) that can be performed. Capturing the correct data is crucial for successful quantitative analysis later in the process.

**Fig 2:**
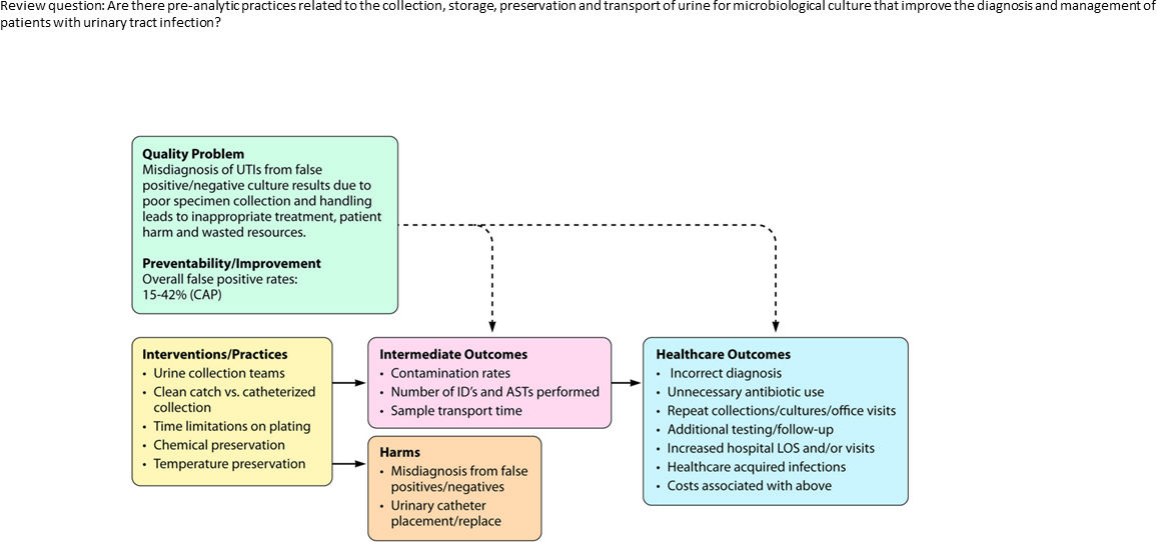
Analytic framework. Adapetd from LaRocco et al. (2). Review question: Are there pre-analytic practices related to the collection, storage, preservation, and transport of urine for microbiological culture that improve the diagnosis and management of patients with urinary tract infection?

LaRocco and colleagues provide a simplified example (Fig. 2) of an analytic framework (2). Several aspects of the LaRocco analytic model are worth noting: (i) The single model gave rise to eight different practice (evidence analysis) questions. (ii) The model links the eight questions into a coherent framework to build the evidence base, piece by piece, within the larger process of interest. (iii) The model suggests many more review questions that could have been asked and may need to be asked in future research. (iv) The model could easily be elaborated and will likely have to be for future projects that seek to integrate the findings in LaRocco work into the complexities of the patient care process (36).

In this framework, the Review Question was defined first, “Are there preanalytical practices related to the collection, storage, preservation, and transport of urine for microbiological culture that improve the diagnosis and management of patients with urinary tract infections?” The quality issue/problem was articulated based on the 15% to 42% false-positive urine culture rates defined by CAP using quality metrics (2). Interventions and practices to answer the review question and address the problem were then listed and, in this case, included the following practices: (i) the use of urine collection teams, (ii) how the clean catch compared with a catheterized collection, (iii) the time limitations from collection to plating, and (iv) whether chemical preservation or refrigeration was necessary. Intermediate outcomes to indicate a successful intervention would be a lowering of the contamination rate, a decrease in the number of identifications and antimicrobial susceptibilities performed, and a reduction in transport time. Harms to the patient also needed to be considered, i.e., the possibility of no treatment because of a false negative result or the wrong therapy because of a false positive result, as well as the potential for unnecessary placement of an indwelling urinary catheter or incorrect categorization of a CAUTI (catheter-associated urinary tract infection) for quality reporting purposes. Finally, general healthcare outcomes were considered. These are related to specific clinical and financial outcomes that should be avoided, including: (i) incorrect diagnosis, (ii) unnecessary antibiotic use, (iii) repeat urine cultures if the physician questions the result, and (iv) the possibility of healthcare-acquired infections and additional healthcare costs.

### ASM’s experience with meta-biostatistician assistance in creating the analytic framework

Comprehensive SR with meta-analysis projects may include questions that encompass the entire laboratory medicine process: from the comparison of pre-analytical practices, intra-analytical practices (including test use based on diagnostic accuracy), and post-analytic practices (including the effect of diagnostic tests on patient management), as well as longer-term individual health and organizational outcomes (19). It was learned through ASM’s collaborative efforts with CDC DLS that it is vital for the SR Team and Expert Panel to have a clear picture of how these different components inter-relate in preparation for the analysis (A-4) step, including quantitative meta-analysis. Meta-analyses can be carried out only when the review question(s) has been clearly and narrowly specified. This approach can sometimes be confusing in a larger and more comprehensive evidence analysis project. Thus, the meta-biostatistician is critical when working with and advising the SR Team and Expert Panel to create the analytic framework.

### ASM’s experience planning for study designs during the preparation of the analytic framework

As clinical laboratories and the entire healthcare system continuously evolve, increasing focus is placed on improving the value of services, including patient and organizational outcomes. As laboratory interventions and diagnostic interventions become more data-driven, outcome-oriented, and evidence-based, laboratory professionals will need to become well-versed in aspects of experimental design, biostatistics, epidemiology, quality improvement science, implementation science, and evaluation science. For instance, understanding the conditions necessary for a high-quality experimental study design, assessment of study bias, and review of confounding experimental variables are critical aspects of well-constructed quality improvement studies. Knowledge of these concepts will allow laboratory scientists to generate data that can be more suitable for inclusion in clinical outcome studies derived from quality improvement projects, comparative analytics studies, or impact studies of new testing methods.

*“In vitro* diagnostic*”* (IVD) clinical research studies that utilize “de-identified” human tissues or fluids, which cannot be linked long-term to a living individual, are commonly performed in collaboration with clinical laboratories that perform research. This type of clinical research differs from what is defined as “patient-oriented research,” which is commonly interventional, also described as “research conducted with human subjects,” and may occur in collaboration with clinical laboratories or outside of the laboratory. To review the various types of experimental designs, formulation of a sound and reasonable hypothesis is always the starting point, followed by appropriate and ethical study design, choice of relevant endpoints, assessment of study bias and confounding variables, statistical analysis, feasibility, and hypothesis testing—for details about the various aspects of research, the reader can refer to several references (19, 36–43).

Given an evolving healthcare system, emerging technology, and expanding competencies required for laboratory professionals, the LMBP Initiative was created to develop a standardized process by which laboratories can identify and evaluate quality improvement practices that effectively improve healthcare quality and patient outcomes. These efforts mainly focus on pre-analytical and post-analytical phases of laboratory testing, where errors occur (19). LMBP created a variation of existing qualitative and quantitative synthesis approaches in Systematic Reviews (41–43) but with the same goal of making laboratory guidelines and study conclusions stronger than any single study’s analysis alone.

**Fig 3:**
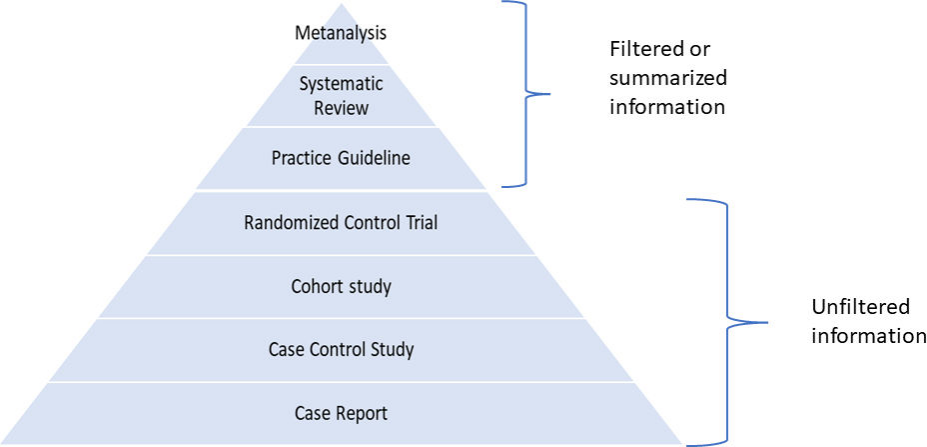
Ranking in terms of scientific rigor, with meta-analysis representing the highest form of scientific rigor.

The ideal approach to synthesis is generally quantitative. Meta-analysis lends itself to the numerical synthesis of the combined studies' effect sizes and requires knowledge of advanced statistical techniques and study populations' heterogeneity. Although meta-analysis is the study type with the highest rigor (Fig. 3) for answering questions, there can still be variation in the confidence of meta-analytic results since there are distinct levels of evidence that provide the basis for the meta-analysis. As noted above, a meta-analysis of high-rigor RCTs would provide the highest level of confidence for individual treatment questions, whereas a meta-analysis of cross-sectional diagnostic accuracy studies would provide the highest level of confidence regarding test accuracy (44, 45). For laboratory process improvement questions, where results are sensitive to highly localized contexts, other types of study designs may be optimal (14).

### ASM’s experience with acquire step – A-2, Keywords and strings

ASM’s use of step A-2 involved the development of a SR search strategy aimed at finding all relevant literature on the topic. Through the ASM-CDC DLS collaboration, it was learned that search strategy development is most successful when there is the involvement of a Medical Librarian, the Expert Panel, and a Meta-biostatistician (descriptions of each of their roles appear in later subsections). It was found that search strategies should be designed with high sensitivity for maximum retrieval of relevant literature. A highly sensitive strategy incorporates an appropriate combination of subject headings and text words. Subject headings are standardized terms used in a bibliographic database’s indexing scheme or controlled vocabulary. MeSH (Medical Subject Heading) terms are assigned to MEDLINE/PubMed records to describe the content and enhance access, as Emtree terms are to Embase records (46). Other bibliographic databases use different schemes to facilitate information retrieval. The searcher can identify and map subject headings using the database’s online thesaurus. ASM additionally discovered that relying entirely on subject headings for SR literature searching is not recommended, as quality and depth of indexing varies across bibliographic records. Subject headings provide a solid foundation on which to build a search strategy and account for indexing shortfalls, text words are added to boost its retrieval potential. Only a few of the earliest LMBP Initiatives (1, 8) sought grey data to avoid publication bias (22). In those studies, library databases that included grey data were used, along with direct calls for data with or without pre-conceived data gathering templates. Since then, ASM excluded grey data; therefore, some publication bias will be a limitation in EBLMPGs published in or after 2019.

During ASM’s adoption of the A-6 cycle method, key concepts were derived from review questions, and analytic frameworks were used to identify terms formed into search strings. It was learned that review questions structured in a standard format, such as PICO components, can quickly be broken down into searchable concepts. Not all concepts are included in the strategy, as certain concepts are challenging to define as search terms. For instance, concepts that refer to specific populations, settings, or outcomes may not lend themselves to searching because they are inadequately described in titles and abstracts and poorly defined in controlled vocabulary (47). It was learned that attempts to capture concepts could inflate or limit the results of a search in undesirable ways. Other concepts, like the type of study, are common to SRs and validated strategies are made available for typical use. These validated strategies are called filters or hedges and are incorporated into a strategy to increase sensitivity. Filters designed for specific databases should be used accordingly. For example, the Cochrane Occupational Safety and Health Review Group has developed three randomized control trial (RCT) filters: one for PubMed, one for Embase.com, and one for PsycINFO ProQuest. Each filter accounts for its own controlled vocabulary, field identifiers, and syntax specific to the database and platform.

A final search strategy evaluation was developed iteratively across targeted literature databases. For example, a broad initial sweep of the literature was launched to find a sample set of relevant citations, from which additional terms and subject headings were harvested for inclusion in the search strategy. Once a draft text of the strategy had been scripted, the searcher tested the usefulness of additional search elements like alternate spellings, truncation characters, adjacency operators, and field restrictions. The search strategy, typically developed for the database, given the highest priority, was modified for subsequent databases to account for variations in database capabilities, structure, and search syntax. The strategy evolved through several iterations, as it was reviewed, tested, revised, and finally approved by the team. The ultimate search strategy treads a delicate balance between being reasonably specific (i.e., minimizing inclusion of irrelevant citations in the search results) and highly sensitive (i.e., maximizing inclusion of relevant citations in the search results).

It was learned that mistakes in the strategy can result in a biased or incomplete evidence base, negatively impacting the quality of the SR (48). For example, some of the most common errors in search strategies include missed MeSH terms, irrelevant terms and subject headings, missed spelling variants, and inappropriate use of logical operators (49). It was also learned that searchers may submit their strategies to colleagues for peer review. A formal peer review process for electronic search strategies has yet to be established; however, a tool called the PRESS Instrument, developed by the Canadian Agency for Drugs and Technology in Health (CADTH) and the Cochrane Information Retrieval Methods Work Group, has demonstrated value in facilitating strategy evaluation (50). This tool identifies the elements of the search strategy most likely to impact the accuracy and completeness of the evidence base (49).

#### ASM’s experience with the role of the medical librarian

IOM standards for Systematic Reviews underscore the need to employ a librarian or information management professional with expertise in searching (51). Medical librarians have unique expertise in managing information related to clinical and biomedical literature sources and are highly educated professionals with a unique set of applicable skills that contribute measurably to a SR overall integrity and credibility. They are studied and practiced in collecting, indexing, storing, and retrieving bibliographic information. They understand the mechanics of searching and the logic of search strings, and they can design complex strategies to achieve search objectives. Furthermore, they are aware of hundreds of databases and information resources that can be used for evidence gathering. They also possess training in numerous database systems and software applications for storing and managing large amounts of information. They maintain professional connections with thousands of knowledgeable librarians with whom they can consult. A medical librarian with specific training in performing SR searches is recommended, as the search methods employed have a different strategy than routine searches. There are librarians who not only have had additional training in the SR process but also have had prior experience working on projects.

Developing rigorous, appropriate, and complete search strategies is a skill honed by experience and practiced daily by librarians, as is detailed record keeping. The quality of searching and reporting is significantly improved when a trained librarian is engaged in the SR process (52). Librarians should be introduced to the SR project in the initial planning phases. Meeting with the team early in the process will help the librarian understand the rationale for the project, the questions being investigated, and the protocol being used. The information gathered at this point can be synthesized to draft a preliminary search strategy with which a pilot search can be launched. The results of this search can conclusively establish the need for a SR and identify any previous reviews published on the topic. The findings can help the team refine or refocus the study questions and protocol.

Experienced SR searchers understand the importance of maintaining reporting standards. The SR methods must be transparent, replicable, and updatable. The search process, strategy, and results must be meticulously documented and fully reported. A failure to accurately record and report on these elements degrades the value of the SR. During the search phase, the librarian keeps track of all reportable elements. At the end of the search phase, the team receives a complete library of retrieved references with all pertinent bibliographic information and the final search report.

To best meet Preferred Reporting Items for SRs and Meta-Analyses (PRISMA) guidelines, the librarian’s final report should include the following: the complete search strategy as it was executed in each database searched; database information for each database searched, including time coverage and platform information; a record of the number of citations retrieved per database; and the date the search was executed (53). These items will then be reported in the methods section of, or an attached appendix to, the final manuscript. Without this information, the SR is neither replicable nor updatable. Copies of search reports must be maintained as a component of the SR process.

If a significant amount of time lapses between the date of the initial search and the date the manuscript is submitted for publication, a search refresh is required. The review team will again employ the librarian to run the update, who will document the update process and report on the necessary elements. With this information, any librarian can perform an update or a refresh of the literature lists. If the complete original search strategies and database information are unavailable, performing the update is impossible.

#### Databases for literature searches

Databases were selected based on subject content, date coverage, and scope. Appropriate database choices will account for the subject in question, the population under study, and the likelihood of finding relevant publications (51). Searchers considered what journals the database indexes and how often it’s updated with new information. It was learned that when making database decisions, secondary factors such as cost, accessibility, and usability may come into play. Though time and money can influence decisions, selection is “ideally based on the potential contribution of each database to the project or on the potential for bias if a database is excluded, as supported by research evidence” (54). In addition to vetted databases, hundreds of information resources are publicly available on association, agency, and government websites. These will provide access to published articles, unpublished papers, technical reports, conference proceedings, grey literature, and government documents. Once electronic resources are exhausted, supplemental hand-searching of subject bibliographies, reference lists, and select journals identifies studies that may have been overlooked.

Based on guidance provided by the CDC Librarian, who has special training in SR processes, it was learned that a minimum of three bibliographic databases should be used to gather the evidence (55). The three most important databases for clinical trials, per Cochrane guidelines, are MEDLINE/PubMed, Embase, and Cochrane Central Register of Controlled Trials (46). MEDLINE and Embase together provide good coverage of the biomedical literature. ASM has also used SCOPUS and CINAHL. For questions that were interdisciplinary in nature, additional searching in subject-specific databases is warranted. Subject-specific and regional databases were searched when the core databases were deemed unlikely to yield a complete evidence base (51), such as PsycINFO, commonly searched to access mental health or behavioral science literature, and CINAHL, used for nursing and allied health. Regional databases were also introduced to capture literature targeting specific populations or settings. Two good examples of regional databases are LILACS, which provides comprehensive coverage of Caribbean and Latin American literature, and African Index Medicus, for literature published in and about Africa.

#### ASM’s experience with the role of the expert panel

The primary role of the Expert Panel in the Acquire Step (A-2) is to provide feedback and input to the SR team for a Laboratory Medicine Best Practices SR. The panel may also contribute to the final manuscript. The panel members should be knowledgeable about the topic to be reviewed and understand evidence review methods and data management. Potential panelists are evaluated and selected based on their publication record and their level of involvement and leadership in relevant organizations and initiatives (19). The Expert Panel composition should be selected before the start of the SR.

#### ASM’s experience with the role of the meta-biostatistician

It is vital to capture (i.e., “Acquire”) the correct information in the right way to conduct the proper analysis. Readers new to the SR/metanalysis process may not be aware that even when considering the same diagnostic test, the information captured (and, hence, data structure) can be very different depending on whether the question is about the accuracy of the test relative to a reference test (i.e., a diagnostic test accuracy question) versus questions about the test’s use and timing in the patient care process as they relate to patient health outcomes (i.e., practice intervention or even prognosis question) (56). For example, if the question is specifically about diagnostic test accuracy, true/false positives, and true/false negatives must be extracted from the individual studies. In contrast, for practice intervention/improvement or prognosis questions, data extraction would include group means, measures of dispersion, odds ratios, hazard ratios, etc. From the perspective of quantitative analysis, not only do these two types of data have very different data structures but very different meta-analytic methods as well (57).

Before data extraction, the Expert Panel and Review Team (with the input of the meta-biostatistician) should identify whether the questions of interest relate to a practice intervention (i.e., comparison of implemented quality improvement practices, protocols, procedures, etc.) or (from an *in vitro* diagnostic perspective) relate to analytic validity, clinical validity, or clinical utility (58, 59). Appropriate study designs for inclusion, the structure of the data collection tool, the methods of analysis, and, ultimately, the conclusions that can be drawn all vary depending on which of the above questions are being asked. These must be specified ahead of time for the project to be successful.

The meta-biostatistician is not merely an expert in computation but should be an expert in the methodological procedures and principles of creating SRs and meta-analyses of reviewed studies at many steps along the process. While the statistical methods involved in most non-diagnostic accuracy meta-analyses are less complicated; therefore, the development of several resources (59, 60) and (Review Manager software RevMan version 5.3; http://community.cochrane.org/tools/review-production-tools/revman-5) allow non-statisticians to quickly and easily compute pooled effect sizes and measures of heterogeneity as well as create forest and funnel plots; there are hazards with these types of meta-analyses, which are easily avoided with periodic consultation with a meta-biostatistician. In contrast, the challenges with diagnostic accuracy meta-analyses are far more complex and require more intensive involvement of the meta-biostatistician. In either case, the participation of a meta-biostatistician versed in the methods of the more extensive evidence analysis process is essential. For either diagnostic accuracy or practice intervention/improvement questions, consultation with a meta-biostatistician is critical regarding issues of confounding, model veracity, and publication bias.

Though a meta-biostatistician should be included in the evidence analysis team, there is no definitive guide for when and how the meta-biostatistician should be included in the evidence analysis process. The issue of when to involve the meta-biostatistician in the more extensive evidence analysis process is important (61). Indeed, the LMBP is an integrated process where activities that occur in any one part of the process are carried out with thoughtful reference to other steps in the LMBP A-6 cycle (19). For instance, formulating practice-relevant evidence analysis questions has direct implications for not only the types of data to be extracted from research studies but also the best (or possible) analyses that can be carried out on these data as well as the limitations of what can be inferred from the evidence. Not having the correct data in the proper format can hinder or even preclude the possibility of analyses of interest for stakeholders.

The role depends as much on the other team members’ experience as the meta-biostatistician’s experiences. In general, however, the involvement of the meta-biostatistician should not be limited only to the ANALYZE step. An experienced meta-biostatistician should be able to offer valuable input at several points in the process (see Table 1). There are at least three key analysis challenges in which meta-biostatistician input is crucial, and these will be discussed in the context of the A-6 step in which they are encountered (Table 4). These challenges include the following:

1. Creating the analytic framework (ASK)
2. Identifying the right information and right structure before analysis (ASK)
3. Addressing the challenges of diagnostic test accuracy meta-analyses (ANALYZE)

**TABLE 4:** Steps in the LMBP A-6 process for input from the meta-statistician

LMBP A-6 step	Input of meta-statistician
ASK (A-1)	Consultation on question formulation, methodological criteria for specifying inclusion and exclusion criteria, creation of the logic model or analytic framework
APPRAISE (A-3)	Input on the structure of data extraction fields tailored to the particular project (to facilitate meta-analyses) Available for technical data extraction questions Consultation on effect size
ANALYZE (A-4)	Help with statistical measure conversions to facilitate meta-analysis Recommendations for dealing with missing data Methods for conducting indirect comparisons and more complex mixed treatment methods. Sensitivity analyses

#### Collecting and storing SR data

The review team developed a data management plan for data collection, management, and storage. A SR literature search’s final publication data set typically contains thousands of citations. The plan established a reference management software program designed to handle the large volume of citations characteristic of SRs. Commonly used software programs include Endnote, RefWorks, Reference Manager, and others (62). ASM learned that it was important that all members of the team needed to be working in the same software program and that a central repository of publications be kept. Modifications to the reference library and records should be visible and shared with the whole team. Operating in multiple programs and funneling results from one system to another could result in lost data, complicating reference tracking and counts.

The commercial bibliographic databases employed in evidence retrieval commonly include a feature to facilitate the exportation of hundreds of bibliographic citations at a time. These databases often allow for the direct export of citations into a reference software program and provide options to download information in importable file formats. For proprietary reasons, the database vendor limits the number of citations that can be exported simultaneously. If the results exceed these limits, citations must be downloaded in batches. Keeping careful track of the numbers during exporting and importing is essential. The smaller information sources may not have citation exportation as an option. Relevant citations identified in these sources are manually entered into the citation library as new records. ASM used RefWorks, but is currently using EndNote.

### ASM’s experience with appraise Step – A-3

Step A-3 involves screening evidence obtained during step A-2 for inclusion or exclusion, generally by a straightforward title or abstract review by at least two team members who agree upon inclusion. Inclusion and exclusion criteria are well-defined, and careful records of title disposition are maintained. For studies deemed acceptable for inclusion, this step is followed by abstracting information from the included evidence into standardized data extraction templates, (formerly called abstraction forms by CDC DLS) with determinations of RoB ratings and effect size measures for individual studies.

#### Translational laboratory medicine studies (diagnostic accuracy)

In clinical laboratory settings, a translational step is to adapt and deploy findings from patient-oriented research or clinical trials into daily clinical practice. These transfer-of-practice studies are typically called Method Comparisons or Diagnostic Accuracy Studies. As an example of how diagnostic clinical trial data may enhance our understanding of infections, multiplex molecular panel testing identified an increased frequency of co-infections that spawned investigations to detail the clinical impact of co-infections on disease severity. For more detail on reporting the results of such diagnostic tests, refer to the current document, “Statistical Guidance on Reporting Results from Studies Evaluating Diagnostic Tests; Guidance for Industry and FDA Reviewers” (40).

#### Incorporating large-scale clinical trials

Most large-scale clinical trials are randomized (RCT) in the pharmaceutical industry but are uncommon in the clinical laboratory (37). Clinical trials are always hypothesis-driven and need to be adequately powered, i.e., large enough to ensure that a negative result is not the result of insufficient samples or patients but an actual biological result or diagnostic condition. For non-RCT, clinical outcomes may not be defined; however, the study size and general study design would be amenable to the LMBP A-6 process if the study answers any of the LMBP questions posed for a particular project.

#### Incorporating other meta-analysis

A “meta-analysis” is the process by which the results of multiple individual studies are statistically combined (41, 42). Meta-analysis is a component of the SR process. It is a method for systematically combining pertinent qualitative and quantitative study data from several selected studies to develop a single conclusion that has greater statistical power. This conclusion is statistically more robust than the analysis of any single study due to increased numbers of subjects, greater diversity among subjects, or accumulated effects and results.

#### Bias in meta-analysis

Because of the complex and time-consuming nature of the SR process, meta-analysis should include mitigation of certain pitfalls. Human literature review and grading processes are inherently subjective, and if not carefully designed and executed, appropriate studies may accidentally be discarded. The infrastructure supporting multiple human raters for each publication reviewed, third-party tie breakers, and strict guidelines for data abstraction help mitigate the biases that could occur. Bias can also occur if the studies pooled for SR are a mixture of different experimental design types (e.g., observational studies mixed with RCT). When mitigating bias, the pooled study types should be similar (i.e., all randomized controlled trials or all observational studies).

Publication bias (also called small study bias) is another type of bias in which studies with positive results have a better chance of being published, are published earlier, and are published in journals with higher impact factors. In addition, studies tend to be published only when there are positive results; therefore, studies with negative results are excluded from the published literature, and conclusions based exclusively on published studies can be misleading. In another example, publication bias could occur because some healthcare settings are not as likely to publish their results (e.g., community hospitals vs. academic medical centers). Publication bias is common in the healthcare literature and may cause readers to understand a problem differently than if they had information about a broader group of healthcare organizations. It is optimal when publication bias is assessed and documented (63), preferable with the help of a meta-biostatistician.

#### Confounding variables in study design

When reviewing primary literature, subject matter experts must identify RoB and use their subject matter expertise to create plausible scenarios to describe how or why the interventions work in a healthcare or laboratory setting to assess the generalizability of the final results. To do that, reviewers must understand the relationship of variables in the context of the quality question, identifying confounding variables in order to control for them. For instance, a confounding variable (a confounding factor or confounder) is an extraneous variable in a statistical model that correlates (positively or negatively) with both the dependent and independent variables, distorting the perceived relationship between variables. Confounding variables can occur in a primary clinical study of infection when an infectious exposure (independent variable) and an outcome like mortality (dependent variable) are both strongly associated with a third (possibly random and unrelated) variable (like end-stage cardiac disease). In contrast, moderator variables are those for which the effect of the predictor on the outcome varies; they specify conditions under which the relationship changes direction. For example, a moderating variable could provide insight into how the intervention may work differently in different circumstances (e.g., Emergency Department versus Critical Care versus general inpatient units). Finally, mediator variables are intermediate variables in a causal chain between two other variables. Within the context of SRs and meta-analysis, in addition to subject matter expertise, confounding variables to detect bias occur via subgroup analyses for studies that do and do not adjust for known confounders (64) and subgroup analysis by diagnostic testing method or device.

It is essential to control for confounding effects and assess the impact of the variables of interest. For any research study, it is crucial to determine whether the designed study will answer the question raised in the hypothesis. In clinical laboratory-based research, the control of confounders may be more easily achieved as the investigator can match samples for many baseline characteristics (e.g., disease states) and have control over the environment during the study. Considering subject age, gender, race, and co-morbidities can help the investigator match subjects in a control and an intervention group. It should be considered during the experimental design phases of a study.

In clinical research, controlling for confounders is often more challenging. For example, the laboratory may have little control over the type of patients who enter an emergency department with sepsis, but the laboratory can record information describing the subjects being tested and compare characteristics between two cohorts after the study period ends. When the effects of confounders are not controlled for, they are often dealt with through statistical adjustment, i.e., the use of regression analyses. However, assessing the impact of confounders by these techniques on study outcomes may still be inadequate (65–67).

#### Specific challenges for bias in diagnostic studies

Many factors impact the reporting of diagnostic accuracy studies. For example, the true gold or reference standard may be defined differently in different studies, depending on the methodology used and the purpose of the study. In many situations in evaluating a diagnostic laboratory test or product, there is either no gold standard or its use is impractical, which can introduce variability in study rankings and potential for bias. In these instances, the ideal would be to calibrate the new test to the known performance of an accepted reference standard; however, this is not always done or possible. Without such calibration, the indiscriminate use of terms such as sensitivity and speciﬁcity could be misleading. In such cases, positive and negative agreement are the preferred terms. In agreement studies, the focus is on evaluating a diagnostic test by assessing its agreement in performance with some other well-understood but imperfect test(s).

In diagnostic test evaluation, there are also situations in which the truth may be known but only for a subset of subjects or specimens. These situations can lead to verification bias. In such cases, adjustment for veriﬁcation bias is imperative because establishing the truth of the patient’s condition depends inherently on the test or tests applied to a small and possibly not representative group of patients. An example of introducing bias would be the use of “discrepant testing,” a resolution of discrepancy in which discordant results between the two methods receive further testing. Although commonly reported, this practice introduces a bias toward confirmation of the new test and is discouraged (68).

In another example, the laboratory investigator often needs to establish the limit of detection (LOD), for which a nonparametric statistical approach can be used. This method is commonly used in statistics to model and analyze ordinal or nominal data with small sample sizes (69). Different ways of determining LOD exist. For example, some use 19 positives out of 20 to define the LOD, whereas some use probit analysis. Due to the inherent differences in determining LOD, the RoB must be considered to ensure diagnostic accuracy. To control for diagnostic study bias, the use of the STARD criteria “Statistical Guidance on Reporting Results from Studies Evaluating Diagnostic Tests; Guidance for Industry and FDA Reviewers” (40) or the GRADE (Grading of Recommendations, Assessment, Development, and Evaluation) tools can be helpful.

#### Abstraction training and performance, the role of an expert panel, and meta-biostatistician

Individuals who volunteered for the Expert Panel underwent structured training sessions using the database designed to enter the abstractions. Each member of the Expert Panel independently abstracted a selected publication, after which a conference call was convened to compare the results of the individual quality ratings. This training helped ensure consistency among team members performing abstractions.

Using the LMBP method, two separate abstractors reviewed each paper. If they disagreed with each other’s assessment, a third person was asked to adjudicate the differences. By the time, this phase was finished, at least 2 of 3 abstractors had agreed on the essence of the evidence summary and placed results in an Evidence Summary Table (EST). The agreement between at least two abstractors was usually honored but, if necessary, was discussed further with other Expert Panel/Review Team members, including the Meta-biostatistician, to audit the process and confirm consensus. ASM recruited 10 to 12 subject matter experts (SMEs) or more, as necessary, who served as the abstractors/Expert Panel and Core Team. The final recommendations were presented to the LMBP Workgroup convened by CDC DLS, which independently assessed the data acquisition, analysis process, and the resultant practice recommendations (15).

### ASM’s experience with the analyze step – A-4

The Analyze step involves aggregating the body of evidence to derive summary findings, including practice recommendations based on a practice’s effect as observed in the evidence base and the quality of evidence, as described in Christenson et al. 2011 (3).

#### Role of the expert panel

Utilizing the LMBP process, the SR team works with the Expert Panel and the meta-biostatistician to assess the strength of evidence for the practice(s) being evaluated and translates the evidence into draft evidence-based recommendations, which are then submitted to the LMBP Workgroup for independent external review (16). After the data are abstracted and analyzed by the meta-biostatistician and reviewed and discussed by the Core Team Members, further discussion ensues about whether recommendations regarding the initial questions can be answered and what potential harms or limitations exist. Then, the Technical Assessment Team, the Technical Review Team, and if available, the outside Stakeholders weigh in. Finally, these recommendations are presented to the LMBP Workgroup by the Review Coordinator and/or Technical Lead and the Meta-biostatistician. Translating summary findings into draft evidence-based practice recommendations includes direct input on recommendation categorizations and the degree of confidence that the practice will do more good than harm, in light of the evidence on both effectiveness and aspects of implementation.

#### Role of the meta-biostatistician

Meta-analytic methods for questions of intervention, etiology, or prognosis are well established. Readers familiar with standard statistical procedures used in primary research (original collection and analysis of data not collected before; secondary research involves examination of data collected and reported in previous studies) should find the computation and interpretation of most non-diagnostic accuracy meta-analytic statistics reasonably straightforward. They are simply extensions of the statistical procedures found in primary research. More recent methods to allow for indirect comparisons (70, 71) and manage heterogeneity (72) have been developed but are not yet widely used and will not be discussed here. Special statistical considerations arise with diagnostic test accuracy (DTA) questions. Four are particularly important:

##### Paired outcome measures

In non-DTA questions (e.g., therapy or prognosis questions), there is typically only one measure of overall effect (e.g., the pooled mean or odds ratio). In DTA questions, two related values are typically reported: sensitivity and specificity, positive and negative predictive value, or positive and negative likelihood ratio. It is recommended that meta-analyses be carried out on all three of these measures (73). While single-value summary statistics are available (e.g., diagnostic odds ratio [DOR]), they may not be clinically useful (74).

##### Outcome measures are related

Because sensitivity and specificity are related (e.g., one typically increases as the other decreases) at different index test threshold levels, their pooled estimates should not be computed separately (65). This dependence means that special statistical modeling procedures are warranted (57).

##### Threshold values

Since a diagnostic test aims to differentiate people with the disease or condition from those who do not, the outcome is binary. Therefore, the index test’s threshold (or cutoff) level is used to “sort” subjects into those with the condition or disease from those without. Changing the threshold level changes the results. Primary DTA studies often evaluate different index test threshold levels, which poses a challenge when combining summary statistics across studies. Sensitivity and specificity values from two primary studies that used very different threshold levels are not easily comparable.

##### Reference test error

When a “gold standard” aka “reference standard” test exists (i.e., where we can be relatively confident that the subject did or did not have the disease or condition), then the ability to determine whether the index test accurately sorts subjects into positive and negative disease (or condition) categories is straightforward (74). However, when the reference test is known to be imperfect or the new or index test is believed to be better than the reference test, then estimates of diagnostic accuracy statistics will not be trustworthy (74).

In summary, unlike treatment, etiology, or prognosis questions, diagnostic test accuracy questions typically have two primary statistics of interest: sensitivity and specificity (along with related positive and negative likelihood ratios). Since sensitivity and specificity are related, standard meta-analysis methods are not warranted, and more complex statistical models must be used to complete the meta-analysis (57, 66). Finally, when the reference test is known to be inaccurate, additional adjustments in the meta-analytic strategy may be warranted (73). All of these considerations speak to the importance of the involvement of the meta-biostatistician in the evidence analysis project. Additionally, because the statistical approach will depend heavily on substantive theoretical and clinical considerations (67), continuing dialog between the meta-biostatistician and the rest of the team is vital throughout the analysis process.

### Presentation and interpretation of meta-analysis, basic points

In the following sections, a brief overview is provided of (i) common methods of presenting pooled effect estimates in meta-analyses, (ii) cautions for interpreting these effects, and (iii) a brief description of heterogeneity in meta-analyses.

### What is a summary or pooled effect?

The heart and soul of a typical meta-analysis is the “summary” (19) or “pooled” measure of effect (75), for example, the estimates of both sensitivity and specificity are obtained when the sensitivity and specificity across all studies in the meta-analysis are combined. The summary measures from each study are weighted to account for differences within the studies. Studies with lower within-study variances are given greater weight than studies with higher within-study variances, and then, these weighted measures are combined. Again, this pooled effect is, ideally, a measure of the “true” effect in the population of interest. Readers interested in more detail about specific pooled effects commonly used in diagnostic accuracy meta-analyses are encouraged to consult the Cochrane Handbook for SRs (76) and other sources (58, 77–79).

Summary effects are typically reported in one of two ways: graphically or as statistical point estimates. As we pointed out above, because of the paired nature of diagnostic accuracy measures (e.g., sensitivity and specificity) and because the tradeoff between these paired measures depends on threshold levels (which may vary both within and between studies), the recommended method of estimating pooled effects in diagnostic accuracy studies (57, 79) is to use hierarchical modeling procedures [either the Bivariate model (79), or HSROC]. HSROC stands for Hierarchical Summary Receiver Operating Characteristic Curve, which calculates diagnostic accuracy models (79). Subsequently, the statistical and graphical reporting of the results for diagnostic accuracy studies (where a gold standard reference test is available) will differ somewhat from clinical laboratory-related meta-analyses that examine more typical outcome measures (like mean differences or odds ratios). Examples of non-diagnostic test accuracy (e.g., pre-analytic practices and diagnostic test accuracy meta-analytic results) are provided in the following section.

### Brief example of non-diagnostic accuracy summary statistics

**Fig 4:**
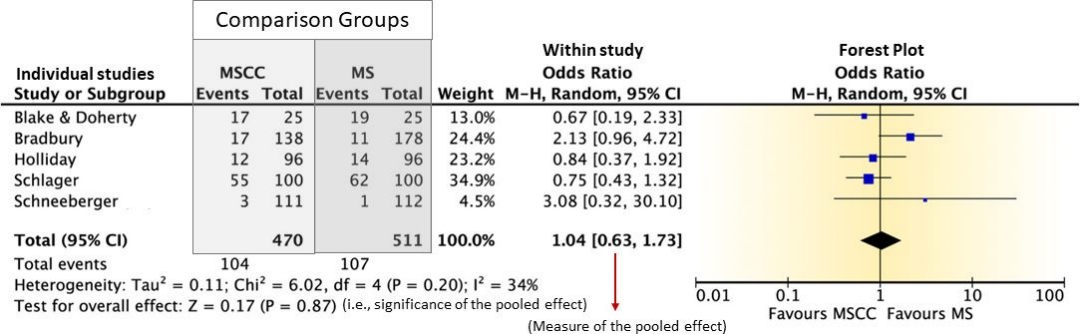
Example of forest plot format, adapted from LaRocco et al. (2). In the figure, each row in the table represents the summary of statistics from each study. Typically, these are organized into comparison groups (arms). The diamond indicates the location of the pooled effect, and the vertical line is the line of equivalence between groups; if the diamond touches the line of equivalence, then the groups are not statistically different. (i) Pooled statistics (the pooled odds ratio) are found at the bottom of the table or below a group of studies if the studies are subgrouped by characteristic. (ii) Heterogeneity (variation between studies not due to chance) statistics are also at the bottom of the figure. (iii) The forest plot (on the right side) is a visual representation of the values on the left portion of the figure. Each study is represented by a box where the size of the box represents the study’s weight, and the whiskers to each side of the box represent within-study error. (iv) The diamond represents the value of the pooled effect size. The width of the diamond indicates the error in the pooled effect measure. (v) Typically, a line of equivalence will be placed down the middle of the forest plot. This line represents the value at which the comparison arms are perfectly equivalent (1 in odds ratios, 0 in comparison of arm means). (vi) When the comparison groups differ on the outcome measure, the pooled effect diamond will fall to one side or the other of the line. (vii) If the pooled effect diamond does not touch the line of equivalence, then the effects in the two comparison groups (arms) are significantly different. The references in the figure are Blake & Doherty (84), Bradbury (85), Holliday (86), Schlager (87), and Schneeberger (88).

Typically, in non-diagnostic test accuracy meta-analyses, measures of effect (e.g., mean difference, odds ratios, Cohen’s d, etc.) are combined across studies in a relatively straightforward manner. Each study contributes to the pooled or summary effect based on a weighting scheme (typically, the inverse of the within-study variance). These pooled effects are then reported, and results are generally presented in a forest plot (see Fig. 4 for example). Importantly, heterogeneity statistics (variation between studies not due to chance) are listed at the bottom of the figure. Both tau^2^ and Higgins I^2^ (80) statistics are generally reported along with the significance of the measure of heterogeneity. Higgins I^2^ is commonly reported as it has a straightforward interpretation as the proportion of total variance due to between-study variation. There are limitations with the I^2^ statistic, however, and careful interpretation is needed (81). There are several free platforms to carry out non-diagnostic accuracy meta-analyses (82, 83). The formatting options will differ slightly for forest plots, but the same basic components and layout described above will be present.

### Examples of common diagnostic accuracy summary statistics

Below, we illustrate the results of meta-analyses by drawing on the analyses reported in LaRocco et al 2016 (2) and based on sample data available from the Cochrane Handbook for DTA reviews (available at http://dta.cochrane.org/handbook-dta-reviews) (47) and published initially in Nishimura (89). STATA 14 was used for the meta-analyses (90). In the examples below, we assume the reader understands the statistical measures of diagnostic accuracy.

### Diagnostic sensitivity and specificity

**Fig 5:**
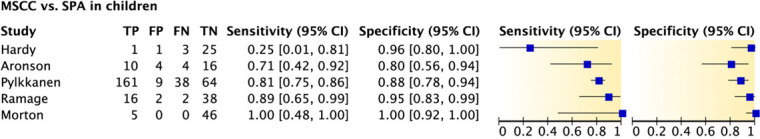
Diagnostic accuracy of midstream clean-catch (MSCC) versus suprapubic aspiration (SPA) for the diagnosis of urinary tract infections in children. Adapted from LaRocco et al. (2). The references in the figure are Hardy (91), Aronson (92), Pylkkanen (93), Ramage (94), and Morton (95).

Sensitivity and specificity for separate studies may be presented graphically (See Fig. 5), as illustrated in the excerpt from LaRocco et al. (2). Figure 5 shows a meta-analysis of the relative diagnostic accuracy of midstream clean-catch (MSCC) versus supra-pubic aspiration (SPA) for the diagnosis of urinary tract infections in children. As with all forest plots, each row represents data from separate studies. Raw data on true/false positives (TP, FP) and true/false negatives (TN, FN) are presented, followed by sensitivity and specificity estimates from each study. On the far right are the forest plots indicating the point estimates of sensitivity and specificity for each study (the blue boxes) and the error bands (the lines to either side of the boxes). A value of 1.0 (as seen in the Morton study) indicates perfect sensitivity or specificity. Notice that there is no line of equivalence.

Of note for the previously described forest plots depicting sensitivity and specificity, pooled effect measures of these two accuracy statistics are not included. These must be computed using the hierarchical models mentioned above. Hence, the purpose of forest plots of sensitivity and specificity is primarily to give the reader a graphical sense of the findings of the different studies included in the analysis, not to provide pooled effect sizes.

Because summary measures of paired statistic pooled effect sizes are computed using hierarchical models, these are typically reported in narrative or tabular format. For a graphic representation of these summary estimates (across different threshold levels), a hierarchical summary ROC curve (HSROC) rather than a forest plot is used (see Fig. 5). Figure 5 is an example table of the pooled effect size estimates from data reported in a review (89) of anticyclic citrullinated peptide antibody (antiCCP) compared with the reference standard for rheumatoid arthritis. We use the Nishimura data rather than the data reported in LaRocco for illustration since the hierarchical models failed to converge (i.e., find a solution) because of the small number of studies in LaRocco (2)—a common limitation when using these more complex models.

We can see from Fig. 5 that the pooled specificity (.96, 95% CI 0.94–o.97) is higher than sensitivity (.66, 95% CI 0.60–0.71) when using anti-CCP to diagnose rheumatoid arthritis compared with the reference standard. The pooled diagnostic odds ratio (DOR) of 43.05 (95% CI.32.00–57.93) is an unpaired measure of test effectiveness, indicating that a subject with the disease is 43.05 times more likely to test positive on the anti-CCP test than a subject without the disease is to test positive on the anti-CCP test (89). The positive and negative likelihood ratios (LR+ and LR−, respectively) indicate how much more likely (LR+) a patient who has a positive diagnosis according to the anti-CCP test is to be a True Positive than a False Positive (15.39 times more likely) and how much more likely (1/LR−) a patient who has a negative diagnosis according to the anti-CCP test is to be a True Negative than a False Negative (2.8 times more likely). A diagnostic test should have an LR+ and a 1/LR− of at least 10 to be considered clinically useful to rule in and rule out (respectively) diagnoses (96). All of the above estimates pool values across studies, ignoring heterogeneity (80, 97).

### HSROC curves

**Fig 6:**
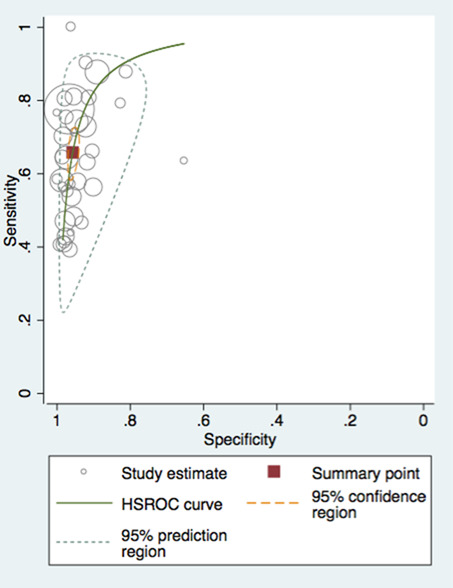
HSROC curve for diagnosis of rheumatoid arthritis using anti‐cyclic citrullinated peptide antibody (anti‐CCP) compared with the reference standard (created using metandi command in STATA 14) (data from reference 89).

As noted above, threshold (cutoff) values for the index test typically vary across studies and often within studies. Because the sensitivity and specificity values will vary by threshold level, one could create a series of forest plots specific to each threshold level. If threshold values vary significantly across studies, then the number of studies for each forest plot may be dramatically reduced. A hierarchical summary ROC curve (HSROC) is an alternative to multiple forest plots. This graphical summary shows the relationship between sensitivity and specificity across a range of threshold values (See Fig. 6).

Figure 6 presents an HSROC graph created using STATA 14 (default metandi command settings). The HSROC curve graph plots sensitivity on the Y-axis against specificity on the X-axis. Note that the X-axis runs from 1 (perfect specificity) at the origin to 0 (perfectly inaccurate for specificity) at the right end of the X-axis. This structure places the location of a perfect index test (the same results as the reference test) at the upper left corner of the graph (i.e., sensitivity = 1 and specificity = 1). Thus, studies closest to the figure’s upper left corner report results that maximize sensitivity and specificity. We describe each of the components of the graph and their utility:

#### Study estimate

Each study included in the meta-analysis is presented as a circle on the graph. The size of the circle corresponds to the weight of the study (different programs will represent weight or relative errors of the within-study sensitivity and specificity in different formats; authors should describe the meaning within the narrative of the meta-analysis). Notice that while most studies cluster closely to the HSROC curve, there are two outliers: one with both low sensitivity and specificity (toward the center of the graph) and one with perfect sensitivity and specificity (located at the upper left corner of the figure). This separation visually separates studies with very different results.

#### HSROC curve

As diagnostic threshold values change, the green solid curve demonstrates the trade-off between sensitivity and specificity. As is typical with ROC curves, specificity begins to decrease as sensitivity increases. At a certain threshold point, specificity decreases dramatically as the proportion of false positives rapidly increases. The summary estimates (Fig. 6) indicate that the anti-CCP test had higher specificity, and this is reflected in the clustering of study estimates (circles) at the left end of the specificity axis (near the origin of the X-axis). In contrast, the sensitivity estimates are more dispersed along the Y-axis, reflecting the lower pooled sensitivity estimate in Fig. 6.

#### 95% prediction region

The region within the dotted blue lines indicates the region within which we can be 95% confident that a future study’s true sensitivity and specificity will be located. This region is a good measure of heterogeneity among studies. A small region indicates low heterogeneity among studies. A large region (like that pictured in Fig. 6) suggests that differences in sensitivity and specificity across studies are likely due to differences between studies (e.g., variation in threshold levels, study design, sample characteristics, implementation methods, etc.). When heterogeneity is high, the model can be further modified to identify potential sources of heterogeneity.

#### Summary point

The red square indicates an average threshold level. As such, it is not clinically meaningful since the optimal threshold level would be the point on the HSROC curve closest to the upper left corner (holding constant the different risks associated with false positive or false negative diagnoses). This point would typically be omitted from HSROC curves, and we include it here only for illustration.

#### 95% confidence region

This is the 95% confidence region around the summary point (average threshold level). As with the summary point, this is a statistical product of the meta-analysis but is not clinically relevant, especially if there is a large variation in threshold levels examined across studies.

Thus, what the HSROC curve adds to the pooled diagnostic effect measures is (i) a picture of the tradeoff between sensitivity and specificity at different diagnostic threshold levels, (ii) a visual way to identify individual studies with very different findings, and (iii) a way of assessing the amount of heterogeneity in the pooled estimates.

### Cautions for interpreting summary effects

There are two fundamental limitations on computing and interpreting a pooled effect size in a meta-analysis: the measures from different studies must be (i) clinically/theoretically comparable and (ii) the same in terms of the measurement statistic. To address the first limitation, before attempting a meta-analysis, it is incumbent upon the content specialists to decide whether the differences between the populations from which study samples are drawn and the pre-analytic procedures, study designs, etc., are sufficiently similar that a summary measure would even make theoretical and clinical sense. For instance, the same diagnostic test may be used differently in adults versus children or may be differentially effective in genetically dissimilar populations (74). This may signal that a single pooled effect size is less meaningful than separate summary effect sizes for these different populations. Changes in pre-analytic practices may also affect the diagnostic accuracy of a test, perhaps indicating separate pooled effects for different pre-analytic practices (98, 99). For intervention studies, the interventions themselves must be sufficiently similar across studies so that quantitatively combining them makes sense. For observational studies, typically used for etiology or prognosis questions, deciding whether measures are similar enough to be combined quantitatively may be challenging. Studies will likely adjust for different confounding variables in these cases, dramatically changing the effect estimate.

To address the second limitation, only outcome measures that are the same across studies can be combined quantitatively to create a pooled effect. In cases where authors do not report the same measures, common measures can be computed or estimated from results reported by the authors. While this may be a less pressing issue in meta-analyses of diagnostic accuracy variables (all derivable from true/false positive and true/false negative values), this becomes a severe limitation in observational studies where very different multivariable models are used and are more common when there is no clear reference test or the reference test is known to be inaccurate (47).

In the above two situations, pooled effect sizes must be interpreted carefully (if pooled effects are calculated at all). Even when pooled effect sizes are calculated, meta-analysis may reveal that differences in estimates between studies are due substantially to systematic differences in study or sample characteristics, that is, heterogeneity. Heterogeneity among studies does not indicate a flaw in the meta-analysis but rather indicates a need for further analysis to identify potential sources of heterogeneity. These analyses are particularly important because the sources of heterogeneity may be of clinical and theoretical importance.

### Measures of heterogeneity

Readers may be familiar with standard measures of dispersion (e.g., standard deviation, standard error, and confidence intervals) in primary research studies. These measures capture the variation of individual subjects or samples around the study outcome (e.g., mean, odds ratio, sensitivity/specificity, etc.). In meta-analyses, there is an additional source of variation around the pooled effect size due to differences in study, test method, and sample characteristics among studies. Hence, in meta-analyses, the dispersion around the pooled effect has two components: within-study (individual level) variation and between-study variation. Measures of heterogeneity in meta-analyses are attempts to quantify the between-study variation.

For instance, no two studies of the same diagnostic test are entirely identical. Study samples, clinical contexts, disease spectrum, study design, patient subgroups, etc., will all vary to some degree between studies. These differences affect pooled effect measures (100). Hence, when attempting to interpret the accuracy of a pooled effect size (in other words, how confident we can be that the pooled effect is an estimate of the “true” population effect), the reader should have some sense of how much of the variance is due to random differences between individuals and how much is due to differences between studies. Producers of meta-analyses should help their readers interpret the heterogeneity measures and continue analyses (where possible) to identify potential sources of heterogeneity.

The last point is an important one. Detection of a high level of heterogeneity does not necessarily signal a problem but often indicates that further analyses are needed to detect the source of that heterogeneity. Differences between sub-populations, procedures, etc., might be evident only from subgroup analysis or meta-regression in non-diagnostic accuracy meta-analyses and from adding covariates to the hierarchical models with diagnostic accuracy studies. These posterior analytic approaches have become very common, but there are some cautions:

- When possible, the evidence analysis team should formulate an *a priori* hypothesis about the effects of potential sources. Performing a series of posterior analyses hunting for possible relationships is not recommended as this increases the likelihood of a type 1 error. When posterior tests are used without an *a priori* hypothesis, the results should be considered only exploratory (58).
- For diagnostic accuracy studies, the evidence analysis team should decide ahead of time, to the extent possible, whether pooled effects or HSROC curves are more appropriate. While the hierarchical bivariate and the HSROC models produce the same results when there are no covariates, they produce different results when covariates are added. Typically, when a common cutoff (threshold) for diagnosis is used, estimates of the pooled sensitivity and specificity are most helpful (hence the bivariate model). When diagnostic cutoff values vary substantially between studies, HSROC curves, and HSROC models are more appropriate (hence the HSROC model) (101).
- A common limitation of heterogeneity analyses is a small number of studies. This limitation is a problem with diagnostic accuracy studies where a small number of studies will result in a situation where the more complex models cannot produce a reliable result, as in Macaskill et al. (57). If heterogeneity analysis is not feasible, the authors should discuss the reasons for this in the narrative.

### ASM’s experience with the apply step – A-5

#### Publication of manuscript

An evidence-based review aims to promote and influence the application of best practices to targeted key stakeholders. Reporting and disseminating evidence-based recommendations through peer-reviewed publications is one avenue for reaching key stakeholders such as laboratory practitioners, laboratory professional organizations, clinicians, administrators, government regulatory agencies, payers, accreditation groups, and policy-makers.

#### Dissemination of information

Healthcare decisions based on scientific evidence have been lacking for many years. In the last 20 years in the United States, significant incentives, or, in some cases, disincentives, have been developed to change the way physicians and other caregivers treat their patients. In all aspects of healthcare, process changes are often challenging to implement. For instance, in hospital epidemiology, compliance with hand washing ranges between 20% and 35% nationally (102). The goal of 90% care based upon evidence by 2020 is discussed in a round table on improved quality of patient care (103).

When a process change is determined to be of benefit, either reducing cost while increasing quality or, in the case of healthcare, improving patient outcomes, the dissemination of the information, and, ultimately, the implementation of the change is often slow to be accepted (104). Many ways of “getting the word out” have been attempted, and the intent of ASM is to disseminate EMLMPG to clinicians, the laboratory, and other stakeholders in an attempt to speed implementation and, thus, improve patient outcomes.

The existence of laboratory guidelines is critical, but if healthcare providers do not know about them, they cannot follow them—and they are of little use. In a survey instituted by Grol et al. 1998 (104), family physicians in the Netherlands had a 67% return of questionnaires representing greater than 1,500 physicians. The dissemination of information and knowledge of the changes were studied; the most reliable method was published in family practice journals at 80%. The next most reliable method was discussing the topic in local physician groups at 53%, and the least reliable method was continuing medical education at 33%. Other forms of dissemination of information include publishing in a journal not specific to the specialty, contact with colleagues, CME course content, and pharmaceutical company information. Other studies (105, 106) include consideration of other groups that may disseminate information on changes in healthcare, such as consumers, healthcare professionals, local administrators, national policy-makers, regulatory bodies, industry, research funders, and researchers (104–106); however, none provide a panacea. More research is required to identify which methods work best to engage physicians for compliance with laboratory guidelines.

Dissemination of information on new technologies and implementing practice guidelines is no easy task. An excellent review of barriers, guidelines, and dissemination strategies can be found in Disseminating Practice Guidelines to Physicians, Quebec, Institut National de Santé Publique du Quebec (https://www.inspq.qc.ca/en/publications/48). Per CDC, all guidelines should be reviewed at least every 5 years and updated to ensure the care provision aligns with current literature.

Identifying barriers, such as time to read and implement changes, has also been discussed (105). Using the single most effective means of information dissemination was compared with multifaceted means of information sharing in several reviews (106, 107) but provides no clear-cut answer on the most effective means of dissemination of information. A major drawback is that only a few included statistical analyses.

#### Knowledge translation of research findings related to accredited laboratories

The long-term goal of ASM is to incorporate EBLMPG guidelines into laboratory best practices and, in some cases, clinical laboratory accreditation checklists to increase compliance with guidelines. The approach is similar to the way that other professional laboratory organizations use revised accreditations standards from deemed status accrediting organizations to justify changes in laboratory medicine practice. Deemed status organizations work as agents for CLIA accreditation inspections to meet compliance requirements for CMS. National organizations with CMS-deemed status include The Joint Commission (TJC), the Commission on Office Laboratory Accreditation (COLA), and the College of American Pathologists (CAP). For example, during inspections by TJC, using patient safety goals to monitor patient outcomes, there is a belief that improved outcomes will result from improved practices—true in some cases (108, 109). The CAP accreditation checklists are a compilation developed over 50 years with input from pathologists and laboratory professionals (110). The checklists contain over 2,000 standards across all laboratory subspecialties, updated yearly. CAP quality programs have improved blood culture methods and other laboratory practices by making them part of their accreditation checklists (111–113).

Likewise, a requirement for a particular best practice in a related regulation or government policy should ensure compliance. Incorporation of best practices into payment policies is yet another mechanism to ensure adoption. ASM supports the view that the publication of high-quality SRs with metanalysis and graded recommendations for practices is a prerequisite to developing and incorporating laboratory best practices into accreditation standards and policy. Without inclusion into accreditation or regulation, guidelines can be ignored or forgotten.

For instance, two practice guidelines published by the National Academy for Clinical Biochemistry on point-of-care testing cover many areas of laboratory testing, including microbiology (20, 114). The guidelines were published in the National Guideline Clearing House and the Library of Congress; it included a listing of the professional societies that contributed to its development. Sadly, one of the documents is now archived due to failure to post any updates in the last 10 years. Since ASM members were involved in this endeavor (20, 114), it became evident that creating a guideline is only the first step. The substantial efforts for guideline creation are optimally followed by efforts to disseminate it and to succeed in changing practices.

Although research is unclear on whether disseminating information is best achieved by choosing one or numerous methods, the evidence-based guidelines from ASM are being distributed via as many avenues as possible. The guidelines are published in clinical microbiology journals, presented at national and local microbiology meetings, and, most importantly, sent to stakeholders from other specialties such as infectious diseases, nursing, and other professional societies.

An overarching goal of the EBLMPG committee of the ASM is the formation and continuation of multidisciplinary teams to develop and disseminate evidence in partnership with governmental agencies such as the CDC, AHRQ, and the National Quality Forum, as well as accrediting agencies, in collaboration with other healthcare professional organizations, such as the American Society for Clinical Pathology (ASCP) and the American Public Health Laboratories (APHL). Other healthcare team members must be included, including physicians, nurses, pharmacists, and other healthcare specialists and their respective professional organizations. The intent is to facilitate the best use of newer guidelines based on evidence in the literature to improve patient outcomes.

### ASM’s experience with the assess step – A-6

Moving available research evidence to the forefront of clinical practice demands a collaborative and systematic approach. It requires exhaustive searches for research information, with appraisal, synthesis, and distillation of the identified/acquired research and its data. The data must then be collected from each study or report and synthesized into organized findings and evidence-based guidelines, often contingent on clinicians’ and scientists’ expertise and experience (Center for Evidence-Based Medicine, https://www.cebm.net/). These guidelines are then disseminated for uptake into clinical practice and standards of care.

The contributions began with the efforts of Professional Laboratory Organizations. The American Association for Clinical Chemistry (AACC) was the first clinical laboratory society to publish laboratory practice guidelines. Initially, the National Academy of Clinical Biochemistry (NACB) published guidelines for point-of-care testing (14), which were developed in a manner similar to consensus guidelines. Since then, CAP has started publishing evidence-based guidelines for laboratory medicine that qualify for and are placed in the NGC. The guidelines that have been produced so far focus on anatomic pathology issues (20). The ASM has also started producing evidence-based guidelines that address clinical microbiology laboratory issues. The following sections address the ASM approach to evidence-based guideline production through adoption of the CDC DLS LMBP A-6 cycle methods.

In the fall of 2014, an ASM *ad hoc* Committee on Evidence-Based Microbiology Laboratory Guidelines Assessment (EBMLGA) was formed to provide feedback to the EBLMPG Committee about guidelines prepared under the CDC Cooperative Agreement. The EBLMPG was charged with independently assessing evidence-based practice guidelines in Clinical Microbiology using the Appraisal of Guidelines for Research and Evaluation (AGREE) assessment tool (115), now in its second revision (AGREE II) (115). AGREE was initially released in 2003 and was developed through an international collaboration of guideline developers and researchers as a standardized method to assess practice guideline variability. As described by Brouwers et al. (115), the AGREE II assessment instrument can be used by a variety of groups, including healthcare providers, guideline developers, policymakers, and educators, to develop guidelines following a highly structured methodology that evaluates the rigor of existing guidelines, aids in decision-making for adopting guidelines, and develops a skill for writing rigorous guidelines. The work of the ASM *ad hoc* committee is documented in a recently published paper (116).

Briefly, the AGREE II instrument covers questions in six different domains (115, 117, 118). These are part of Appraisal of Guidelines 2013 and include (i) scope and purpose (three questions), (ii) stakeholder involvement (three questions), (iii) rigor of development (eight questions), (iv) clarity of presentation (three questions), (v) applicability (four questions), and (vi) editorial independence (two questions). Domain 1 focuses on the guideline’s overall aims, the health questions specifically being addressed, and the targeted population for the guideline. Domain 2 assesses the stakeholders and intended users involved with guideline development. Domain 3 addresses the process and methods used for guideline development. Domain 4 focuses on how well the guideline is written. Domain 5 focuses on implementation issues for the guideline and resources needed for applying the guideline. Domain 6 addresses editorial independence and potential bias. Finally, there is an overall assessment by the reviewer(s) on the quality of the guideline (lowest to highest quality) and recommendations (yes, yes with modifications, and no) for using the guideline by the intended audience/population.

Two guidelines were initially chosen for review by the EBMLGA *ad hoc* committee. The first guideline was on the effectiveness of practices to reduce blood culture contamination, developed under contract with the CDC (5). For comparison, a second guideline developed by the Emergency Nurses Association (ENA) on preventing blood culture contamination was also reviewed by the EBMLGA committee (119). Both guidelines were selected because they were listed on the National Guideline Clearinghouse (NGC) website (which was discontinued in 2018). Guidelines published on the NGC website had to meet specific criteria for inclusion and must have been published within the past 5 years. Before reviewing these guidelines, the committee underwent AGREE II training using an online training module in the resource center at the AGREE website (3).

As previously listed, the first guideline the committee reviewed was on practices to reduce blood culture contamination (4, 19). Each *ad hoc* committee member reviewed the guideline independently, and then, scores were collated. The second guideline from the ENA (119) examined 10 different pre-analytical variables involved in the blood collection process with some overlap with the Snyder et al. review (5). When comparing the domain scores for both guidelines by the *ad hoc* committee, it was clear that there were gaps in both guidelines and some differences in conclusions. The existence of two guidelines on a similar subject, prepared by two different organizations with similar but potentially conflicting recommendations, is not uncommon, and approaches to using several guidelines have been published (120). Of note was that none of the *ad hoc* committee members were familiar with either of the guidelines before joining the committee. Even though both guidelines and their recommendations might directly impact clinical microbiology laboratories, phlebotomy teams that collect blood cultures, or other key stakeholders involved in the process, it was unclear how the results from the two groups were disseminated following their publication to key stakeholders. There are many aspects of guideline implementation; these are too complex to be discussed in this section. As Shekelle et al. (121) pointed out, how guidelines are disseminated is quite variable, and there is often a disconnect between the guideline developers and those responsible for implementing guidelines.

Weaknesses were found in one or both guidelines based on the AGREE II review. We found that some of the AGREE II questions were sometimes difficult to assess or were not applicable to our assessments. One key assessment criterion is that the development group includes individuals from all relevant professional groups. The ENA guideline appeared to nly be developed by emergency nurses without the involvement of microbiologists, phlebotomists, or physicians. Another area where both guidelines could improve is an attempt to obtain a patient viewpoint. Discussions of financial and resource implications were not detailed sufficiently in either guideline. Also, the lack of details about developing audit metrics and ongoing monitoring were weaknesses of both guidelines.

As part of the initial guideline assessment, the committee chair reached out to the ENA leadership to determine whether they would be interested in hearing about our review and consider a mechanism(s) to harmonize some of the findings in future updates of the guidelines. Fortunately, ENA leadership involved with guideline development was very interested in discussing the reviews and hearing more about our process. A conference call with ENA guideline leadership was conducted a few months after the initial review, and ASM shared a summary of our AGREE II findings and proposed that we continue discussions near the time when the ENA guideline update is scheduled. ASM is hopeful that a strong collaboration with other stakeholders, such as ENA, will be established as we move forward with evidence-based practice guidelines for a coming update of the blood culture collection document and will lead to more widespread adoption of guidelines in the future.

Following a 10-month period after the initial assessments, the *ad hoc* committee re-convened to review two newly published best practices reviews (1, 2). These were two best practices SRs with meta-analysis on the effectiveness of practices to increase timeliness of providing targeted therapy for inpatients with bloodstream infections (1) and the effectiveness of preanalytical practices on contamination and diagnostic accuracy of urine cultures (2). Both guidelines were of high quality, and the committee noted some gaps for improvement in subsequent updates. The lack of a patient stakeholder (on the Expert Panel or Workgroup) is one gap.

## EXAMPLES OF SYSTEMATIC REVIEWS

Table 5 is a summary of the published SRs with ASM participation. Links to the publications are included in the table. A summary of CDC Systematic Evidence Reviews is found at (3).

**TABLE 5:** LMBP activity summary^*a*^

Topic (method)	Publications screened (date range)	Number that met quality criteria	Findings	Related statistics	Reference	Revision in progress?
Point of care testing (A-6 method)	unknown	unknown			(20)	No
Blood culture contamination (A-6 Method)	456	26	using venipuncture over intravenous catheter collection using phlebotomy teams to collect blood cultures over the use of non-laboratory staff drawing blood	mean odds ratio of 2.69; 95% CI: 2.03–3.57 mean odds ratio of 2.58; 95% CI: 2.07–3.20	(5)	Yes
Rapid Diagnostics blood cultures (A-6 Method)	1827 (1990–2011)	16	No recommendation is made for or against the use of the three assessed practices of this review due to insufficient evidence. Main results. Rapid molecular testing with direct communication significantly improves timeliness compared to standard testing. Rapid phenotypic techniques with direct communication likely improve the timeliness of targeted therapy. Studies show a significant and homogeneous reduction in mortality associated with rapid molecular testing combined with direct communication.	NA	(1)	Yes
Urine culture, pre-analytic factors (A-6 method)	5,092	35	Practice efficacy and effectiveness were measured by two parameters: reduction of urine culture contamination and increased accuracy of patient diagnosis of urinary tract infection.		(2)	No
*C. difficile* (A-6 adapted to QUADAS tool)	11,222	72			(4)	

^*a*^ A-6 Method = four study quality dimensions equal 10 points and result in three quality ratings, i.e., good, fair, or poor. Cdifficile EBLMPG switched to an adapted QUADAS tool to assess risk of bias in diagnostic accuracy studies within the systematic review.

## FUTURE CHALLENGES

**Fig 7:**
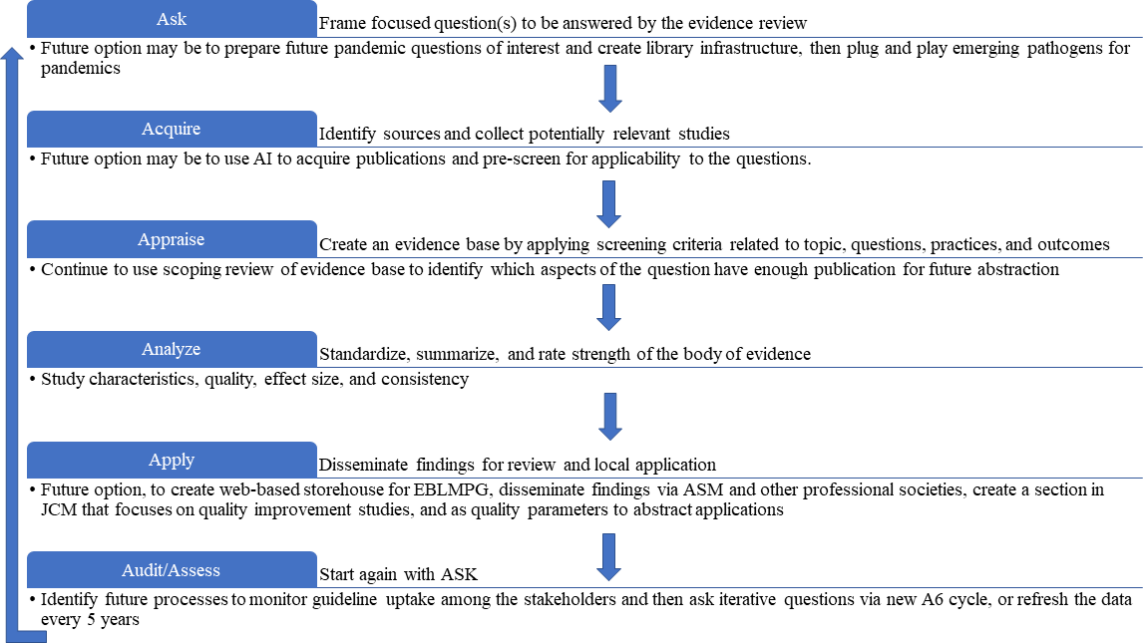
A-6 method adaptations by ASM.

As with any iterative quality or evidence-based process, there are outstanding challenges for the future of EBLMPG. At the minimum, there remains a lack of outcome-based studies to inform the SRs and meta-analysis for other pressing quality questions. Most importantly, although often unfunded, quality improvement activities need to be published more often, since grey data are now eliminated from the process, and patient advocates no longer participate (Fig. 7). There is a large risk for publication bias for quality projects if this change does not occur. A key barrier to the publication challenge is that simple, but important quality reports do not always fit with journal criteria for publication; therefore, it will be prudent for ASM and other professional laboratory organizations to create a space for publishing quality improvement reports or creating a list of journals that accept quality reports and socializing lists with their constituency.

A second barrier to publishing quality improvement studies is the need for more education delivered to the professional laboratory community to ensure that publications comply with current writing standards. The lack of quality publication hinders and slows the EBLMPG process and is critical to future success, for instance, the STARD criteria for diagnostic accuracy and SQUIRE criteria for process improvement studies. Clinical laboratory journals could improve by creating a category for STARD criteria and SQUIRE criteria, thus expanding awareness of these and other publication guidelines so that publications are more likely to achieve the quality ratings required by SRs. For creators of SRs, authors are encouraged to adhere to AMSTAR 2 and PRISMA writing guidelines. With more laboratorians aware of and publishing in these categories, the ability of ASM to speed the guidelines will improve.

ASM made several iterations of LMBP to speed the microbiology process, as details in this review; however, the automation and specialization introduced will not fix the speed problem unless there are more high-quality publications from the laboratory community, which can be collected for SRs to answer questions in the fast-moving space of clinical microbiology and laboratory medicine. Aligning with other professional organizations on this topic for seminars in biostatistics and experimental design for quality projects may help fill this gap. Socializing educational opportunities connected to the US-based Cochrane Reviews and the UK’s Center for Evidence-based Guidelines (CEBM) will be helpful. Finally, improving guidelines for submission of professional meeting abstracts to include GRADE and STARD criteria would help improve abstract quality, which could lead to an easier translation of abstracts to high-quality publication.

While all professional organizations struggle to refresh their practice guidelines, the new EBLMPG practices should make it easier to mitigate the challenge of refreshing the guidelines, with software and automation to speed the review process for the ASM volunteers and the Rutgers staff. Still, the need for rapid reviews, such as those that would have been helpful during the COVID-19 pandemic, is an opportunity for ASM. More pandemics are in the laboratory’s future; therefore, further iterations to rapidly gather publications related to pandemic questions by the librarian, and emergency review process with assessment as early as possible in the pandemic would be optimal. The use of the need for rapid reviews, such as those which would have been helpful during the COVID-19 pandemic, is an opportunity for ASM. More pandemics are in the laboratory’s future; therefore, further iterations to rapidly gather publications related to pandemic questions by the librarian, and emergency review process with assessment as early as possible in the pandemic would be optimal. The use of artificial intelligence with some human assessment in an emergency setting might be used to cull the literature to only the question at hand for a first draft and then refreshed after the pandemic.

Creatively planning this process for future pandemics when the bandwidth of all clinical microbiologists is limited will be a true challenge but would be extremely valuable. Additionally, incorporation of software programs that track quality metrics and pre-prescribed data visualizations that map to clinical and laboratory quality data might be useful. Examples might be Premier or Vizient, among others. However, formal collaborations would need to be established with ASM and those vendors.

Finally, without the collaboration with CDC, ASM’s ability to communicate practice guidelines is limited to their membership and that of other professional collaborators. This approach would likely miss many hospital systems whose leaders do not subscribe to clinical microbiology publications. Perhaps, an opt-in approach to free email lists housed by ASM and an active social presence of the option weblinks for updates would be helpful in circulating the information and recording the uptake of guidelines. Also, working with organizations like CLSI, TJC, and CAP to place guidelines compliance into their checklists would improve adoption.

## INTERESTED IN PARTICIPATING?

The review aims to document the history of EBLMPG, educate laboratory stakeholders about the biostatistics used in laboratory publications and metanalyses, and pique interest in participating in one of ASM’s evidence-based projects. Reading the recent ASM publications in Clinical Microbiology Reviews (1, 2, 4) is a place to begin. More information on the process is available at (3). All volunteers are trained to extract data before participating in any specific project. As the future of laboratory medicine, including clinical microbiology and value-based care, will ultimately be evidence-based, laboratorians and stakeholders are invited to participate. Several roles exist for those who adopt the published guidelines or publish data for a future SR refresh, including trainee, expert panelists, or study site participants.

## References

[1] Buehler SS, Madison B, Snyder SR, Derzon JH, Cornish NE, Saubolle MA, Weissfeld AS, Weinstein MP, Liebow EB, Wolk DM. 2016. Effectiveness of practices to increase timeliness of providing targeted therapy for inpatients with bloodstream infections: a laboratory medicine best practices systematic review and meta-analysis. Clin Microbiol Rev 29:59–103. doi:10.1128/CMR.00053-14 26598385 PMC4771213

[2] LaRocco MT, Franek J, Leibach EK, Weissfeld AS, Kraft CS, Sautter RL, Baselski V, Rodahl D, Peterson EJ, Cornish NE. 2016. Effectiveness of preanalytic practices on contamination and diagnostic accuracy of urine cultures: a laboratory medicine best practices systematic review and meta-analysis. Clin Microbiol Rev 29:105–147. doi:10.1128/CMR.00030-15 26598386 PMC4771218

[3] Christenson RH, Snyder SR, Shaw CS, Derzon JH, Black RS, Mass D, Epner P, Favoretto AM, Liebow EB. 2011. Laboratory medicine best practices: systematic evidence review and evaluation methods for quality improvement. Clin Chem 57:816–825. doi:10.1373/clinchem.2010.157131 21515742

[4] Kraft CS, Parrott JS, Cornish NE, Rubinstein ML, Weissfeld AS, McNult P, Nachamkin I, Humphries RM, Kirn TJ, Dien Bard J, Lutgring JD, Gullett JC, Bittencourt CE, Benson S, Bobenchik AM, Sautter RL, Baselski V, Atlas MC, Marlowe EM, Miller NS, Fischer M, Richter SS, Gilligan P, Snyder JW. 2019. A laboratory medicine best practices systematic review and meta-analysis of nucleic acid amplification tests (NAATs) and algorithms including NAATs for the diagnosis of Clostridioides (Clostridium) difficile in adults. Clin Microbiol Rev 32:e00032-18. doi:10.1128/CMR.00032-18 31142497 PMC6589859

[5] Snyder SR, Favoretto AM, Baetz RA, Derzon JH, Madison BM, Mass D, Shaw CS, Layfield CD, Christenson RH, Liebow EB. 2012. Effectiveness of practices to reduce blood culture contamination: a laboratory medicine best practices systematic review and meta-analysis. Clin Biochem 45:999–1011. doi:10.1016/j.clinbiochem.2012.06.007 22709932 PMC4518453

[6] Sackett DL, Rosenberg WM, Gray JA, Haynes RB, Richardson WS. 1996. Evidence based medicine: what it is and what it isn't. BMJ 312:71–72. doi:10.1136/bmj.312.7023.71 8555924 PMC2349778

[7] McKibbon KA. 1998. Evidence-based practice. Bull Med Libr Assoc 86:396–401. 9681176 PMC226388

[8] Snyder SR, Favoretto AM, Derzon JH, Christenson RH, Kahn SE, Shaw CS, Baetz RA, Mass D, Fantz CR, Raab SS, Tanasijevic MJ, Liebow EB. 2012. Effectiveness of barcoding for reducing patient specimen and laboratory testing identification errors: a laboratory medicine best practices systematic review and meta-analysis. Clin Biochem 45:988–998. doi:10.1016/j.clinbiochem.2012.06.019 22750145 PMC4518452

[9] Agency for Healthcare Research and Quality. 2015. Evidence-based decision making. Rockville, MD

[10] Hallworth MJ. 2011. The ‘70% claim’: what is the evidence base? Ann Clin Biochem 48:487–488. doi:10.1258/acb.2011.011177 22045648

[11] Murad MH, Asi N, Alsawas M, Alahdab F. 2016. New evidence pyramid. Evid Based Med 21:125–127. doi:10.1136/ebmed-2016-110401 27339128 PMC4975798

[12] Hadorn DC, Baker D, Hodges JS, Hicks N. 1996. Rating the quality of evidence for clinical practice guidelines. J Clin Epidemiol 49:749–754. doi:10.1016/0895-4356(96)00019-4 8691224

[13] Shea BJ, Reeves BC, Wells G, Thuku M, Hamel C, Moran J, Moher D, Tugwell P, Welch V, Kristjansson E, Henry DA. 2017. AMSTAR 2: a critical appraisal tool for systematic reviews that include randomised or non-randomised studies of healthcare interventions, or both. BMJ 358:j4008. doi:10.1136/bmj.j4008 28935701 PMC5833365

[14] Brownson RC, Shelton RC, Geng EH, Glasgow RE. 2022. Revisiting concepts of evidence in implementation science. Implement Sci 17:26. doi:10.1186/s13012-022-01201-y 35413917 PMC9004065

[15] Khan KS, Kunz R, Kleijnen J, Antes G. 2003. Five steps to conducting a systematic review. J R Soc Med 96:118–121. doi:10.1177/014107680309600304 12612111 PMC539417

[16] Anonymous. 2011. Institute of medicine (US) committee on standards for developing trustworthy clinical practice guidelines: clinical practice guidelines we can trust. Institute of Medicine, Washington D.C.

[17] Whiting P, Savović J, Higgins JPT, Caldwell DM, Reeves BC, Shea B, Davies P, Kleijnen J, Churchill R, ROBIS group. 2016. ROBIS: a new tool to assess risk of bias in systematic reviews was developed. J Clin Epidemiol 69:225–234. doi:10.1016/j.jclinepi.2015.06.005 26092286 PMC4687950

[18] Perry R, Whitmarsh A, Leach V, Davies P. 2021. A comparison of two assessment tools used in overviews of systematic reviews: ROBIS versus AMSTAR-2. Syst Rev 10:273. doi:10.1186/s13643-021-01819-x 34696810 PMC8543959

[19] Wolcott J, Schwartz A, Goodman C. 2008. Laboratory medicine: a national status report. The Lewin Group, Falls Church, VA.

[20] Campbell SCJ, Hall GS, Lebar WD, Greene W, Roush D, Rudrik JT, Russell B, Sautter R. 2006. Infectious disease laboratory medicine practice guidelines: evidence-based practice for point of care testing. National Academy of Clinical Biochemistry, Washington, D.C.

[21] College of American Pathologists. 2023. CAP guidelines. College of American Pathologists. Available from: https://www.cap.org/protocols-and-guidelines/current-cap-guidelines. Retrieved 19 Dec 2023.

[22] Hopewell S, McDonald S, Clarke M, Egger M. 2007. Grey literature in meta-analyses of randomized trials of health care interventions. Cochrane Database Syst Rev 2007:MR000010. doi:10.1002/14651858.MR000010.pub3 17443631 PMC8973936

[23] Kohn LT, Corrigan JM, Donaldson MS. 2000. To err is human: building a better health system. National Academies Press, Washington, D.C. 25077248

[24] Garg AX, Hackam D, Tonelli M. 2008. Systematic review and meta-analysis: when one study is just not enough. Clin J Am Soc Nephrol 3:253–260. doi:10.2215/CJN.01430307 18178786

[25] Cabana MD, Rand CS, Powe NR, Wu AW, Wilson MH, Abboud PA, Rubin HR. 1999. Why don't physicians follow clinical practice guidelines? A framework for improvement. JAMA 282:1458–1465. doi:10.1001/jama.282.15.1458 10535437

[26] Whiting P, Rutjes AWS, Reitsma JB, Bossuyt PMM, Kleijnen J. 2003. The development of QUADAS: a tool for the quality assessment of studies of diagnostic accuracy included in systematic reviews. BMC Med Res Methodol 3:25. doi:10.1186/1471-2288-3-25 14606960 PMC305345

[27] Whiting PF, Rutjes AWS, Westwood ME, Mallett S, Deeks JJ, Reitsma JB, Leeflang MMG, Sterne JAC, Bossuyt PMM, QUADAS-2 Group. 2011. QUADAS-2: a revised tool for the quality assessment of diagnostic accuracy studies. Ann Intern Med 155:529–536. doi:10.7326/0003-4819-155-8-201110180-00009 22007046

[28] Ouzzani M, Hammady H, Fedorowicz Z, Elmagarmid A. 2016. Rayyan — a web and mobile app for systematic reviews. Syst Rev 5:210. doi:10.1186/s13643-016-0384-4 27919275 PMC5139140

[29] Saldanha IJ, Smith BT, Ntzani E, Jap J, Balk EM, Lau J. 2019. The systematic review data repository (SRDR): descriptive characteristics of publicly available data and opportunities for research. Syst Rev 8:334. doi:10.1186/s13643-019-1250-y 31862012 PMC6925515

[30] Anonymous. 2013. AHRQ systematic review data repository. SRDR: systematic review data repository. Agency for Healthcare Research and Quality. Available from: https://srdrplus.ahrq.gov 32392021

[31] Anonymous. 2009. Systematic reviews: CRD’s guidance for undertaking reviews in healthcare. 2nd ed. Centre for Reviews and Dissemination, York, ENG.

[32] Rubinstein ML, Wolk DM, Babady NE, Johnson JK, Atkinson B, Makim R, Parrott JS. 2021. Mapping the evidence on rapid diagnosis of bloodstream infections: a scoping review. J Appl Lab Med 6:1012–1024. doi:10.1093/jalm/jfab041 34125211

[33] Anderson LM, Petticrew M, Rehfuess E, Armstrong R, Ueffing E, Baker P, Francis D, Tugwell P. 2011. Using logic models to capture complexity in systematic reviews. Res Syn Meth 2:33–42. doi:10.1002/jrsm.32 26061598

[34] Baxter SK, Blank L, Woods HB, Payne N, Rimmer M, Goyder E. 2014. Using logic model methods in systematic review synthesis: describing complex pathways in referral management interventions. BMC Med Res Methodol 14:62. doi:10.1186/1471-2288-14-62 24885751 PMC4028001

[35] Kneale D, Thomas J, Harris K. 2015. Developing and optimising the use of logic models in systematic reviews: exploring practice and good practice in the use of programme theory in reviews. PLoS One 10:e0142187. doi:10.1371/journal.pone.0142187 26575182 PMC4648510

[36] Anderson LM, Petticrew M, Chandler J, Grimshaw J, Tugwell P, O’Neill J, Welch V, Squires J, Churchill R, Shemilt I. 2013. Introducing a series of methodological articles on considering complexity in systematic reviews of interventions. J Clin Epidemiol 66:1205–1208. doi:10.1016/j.jclinepi.2013.07.005 23953080

[37] Seely E, Grinspoon S. 2009. Patient-oriented research: clinical pathophysiology and clinical therapeutics. In Clinical and translational science: principles of human research. Academic Press, San Diego, CA.

[38] Wolk DM. 2016. Clinical and evidence-based research in the clinical laboratory. In Garcia LS (ed), Clinical laboratory management, 2nd ed. ASM Press, Washinton, D.C.

[39] Asher H. 1976. Causal modeling. Sage Publications, Beverly Hills, CA.

[40] Anonymous. 2007. Statistical guidance on reporting resutls from studies evaluating diagnostic tests. U.S. Department of Health and Human Services, Center for Devices and Radiological Health, Washington, D.C.

[41] Anello C, Fleiss JL. 1995. Exploratory or analytic meta-analysis: should we distinguish between them? J Clin Epidemiol 48:109–116; doi:10.1016/0895-4356(94)00084-4 7853037

[42] Chu H, Nie L, Cole SR, Poole C. 2009. Meta-analysis of diagnostic accuracy studies accounting for disease prevalence: alternative parameterizations and model selection. Stat Med 28:2384–2399. doi:10.1002/sim.3627 19499551

[43] Leeflang M, Reitsma J, Scholten R, Rutjes A, Di Nisio M, Deeks J, Bossuyt P. 2007. Impact of adjustment for quality on results of metaanalyses of diagnostic accuracy. Clin Chem 53:164–172. doi:10.1373/clinchem.2006.076398 17185365

[44] OCEBM Levels of Evidence Working Group. 2011. The Oxford levels of evidence 2. Oxford Centre for Evidence-Based Medicine. Available from: https://www.cebm.ox.ac.uk/resources/levels-of-evidence/ocebm-levels-of-evidence. Retrieved 31 Dec 2023.

[45] Howick J, Chalmers I, Glasziou P, Greenhalgh T, Heneghan C, Liberati A, Moschetti I, Phillips B, Thornton H, Goddard O, Hodgkinson M. 2011. The 2011 Oxford CEBM levels of evidence (introductory document). Oxford centre for evidence-based medicine. Available from: https://www.cebm.ox.ac.uk/resources/levels-of-evidence/ocebm-levels-of-evidence. Retrieved 31 Jan 2023.

[46] Gomez O, Taliano J. 2015. Systematic review literature searches - the librarian as a collaborator. The Centers for Disease Controls and Prevention, Stephen B. Thacker Library, Atlanta, GA.

[47] Higgins J, Green S. 2011. Cochrane handbook for systematic reviews of interventions. version 5.1.0. London, England

[48] McGowan J, Sampson M. 2005. Systematic reviews need systematic searchers. J Med Libr Assoc 93:74–80. 15685278 PMC545125

[49] Sampson M, McGowan J, Cogo E, Grimshaw J, Moher D, Lefebvre C. 2009. An evidence-based practice guideline for the peer review of electronic search strategies. J Clin Epidemiol 62:944–952. doi:10.1016/j.jclinepi.2008.10.012 19230612

[50] Relevo R, Paynter R. 2012. Peer review of search strategies. Agency for Healthcare Research and Quality, Rockville, MD. 22787681

[51] Eden J, Levit L, Berg A, Morton S. 2011. Finding what works in healthcare: standards for systematic review. The National Academies Press, Washington, D.C. 24983062

[52] Rethlefsen ML, Farrell AM, Osterhaus Trzasko LC, Brigham TJ. 2015. Librarian co-authors correlated with higher quality reported search strategies in general internal medicine systematic reviews. J Clin Epidemiol 68:617–626. doi:10.1016/j.jclinepi.2014.11.025 25766056

[53] Liberati A, Altman DG, Tetzlaff J, Mulrow C, Gøtzsche PC, Ioannidis JPA, Clarke M, Devereaux PJ, Kleijnen J, Moher D. 2009. The PRISMA statement for reporting systematic reviews and meta-analyses of studies that evaluate health care interventions: explanation and elaboration. J Clin Epidemiol 62:e1–34. doi:10.1016/j.jclinepi.2009.06.006 19631507

[54] Sampson M, Barrowman NJ, Moher D, Klassen TP, Pham B, Platt R, St John PD, Viola R, Raina P. 2003. Should meta-analysts search Embase in addition to Medline? J Clin Epidemiol 56:943–955. doi:10.1016/s0895-4356(03)00110-0 14568625

[55] Centers for Disease Control and Prevention. 2022. Systematic reviews and publications. Centers for Disease Control and Prevention, Division of HIV Prevention, National Center for HIV, Viral Hepatitis, STD, and TB Prevention. Available from: https://www.cdc.gov/hiv/research/interventionresearch/prs/systematic-reviews/index.html. Retrieved 31 Dec 2023.

[56] Normand S-LT, Sykora K, Li P, Mamdani M, Rochon PA, Anderson GM. 2005. Readers guide to critical appraisal of cohort studies: 3. analytical strategies to reduce confounding. BMJ 330:1021–1023. doi:10.1136/bmj.330.7498.1021 15860831 PMC557157

[57] Macaskill P, Gatsonis C, Deeks JJ, Harbord RM, Takwoingi Y, Deeks JJ, Bossuyt PM, Gatsonis C. 2010. Analysing and presenting results. In Cochrane handbook for systematic reviews of diagnostic test accuracy, 1st ed. The Cochrane Collaboration.

[58] Linnet K, Bossuyt PMM, Moons KGM, Reitsma JB. 2012. Quantifying the accuracy of a diagnostic test or marker. Clin Chem 58:1292–1301. doi:10.1373/clinchem.2012.182543 22829313

[59] Thomas J, Graziosi S, Higgins S, Coe R, Torgerson C, Newman M. 2012. Teaching meta-analysis using MetaLight. BMC Res Notes 5:571. doi:10.1186/1756-0500-5-571 23078762 PMC3532381

[60] Wallace BC, Dahabreh IJ, Trikalinos TA, Lau J, Trow P, Schmid CH. 2012. Closing the gap between methodologists and end-users: R as a computaional back-end. J Stat Soft 49:15. doi:10.18637/jss.v049.i05

[61] Parrott JS, Rubinstein ML. 2015. Metacognition and evidence analysis instruction: an educational framework and practical experience. Syst Rev 4:112. doi:10.1186/s13643-015-0101-8 26293452 PMC4546034

[62] Lorenzetti DL, Ghali WA. 2013. Reference management software for systematic reviews and meta-analyses: an exploration of usage and usability. BMC Med Res Methodol 13:141. doi:10.1186/1471-2288-13-141 24237877 PMC3830982

[63] Harrer M, Cuijpers P, Furukawa TA, Ebert DD. 2021. Doing meta-analysis with R: a hands-on guide. 1st ed. Chapman & Hall/CRC Press, London, UK.

[64] Zelig R, Goldstein S, Touger-Decker R, Firestone E, Golden A, Johnson Z, Kaseta A, Sackey J, Tomesko J, Parrott JS. 2022. Tooth loss and nutritional status in older adults: a systematic review and meta-analysis. JDR Clin Trans Res 7:4–15. doi:10.1177/2380084420981016 33345687

[65] Gordis L. 2009. Epidemiology. 4th ed. Saunders/Elsevier, Philadelphia, PA.

[66] Kim KW, Lee J, Choi SH, Huh J, Park SH. 2015. Systematic review and meta-analysis of studies evaluating diagnostic test accuracy: a practical review for clinical researchers-part II. Korean J Radiol 16:1175–1187. doi:10.3348/kjr.2015.16.6.1175 26576106 PMC4644738

[67] Bossuyt P, Davenport C, Deeks J, Hyde C, Leeflang M, Scholten R. 2013. Interpreting results and drawing conclusions. In Deeks JJ, Gatsonis C (ed), Cochrane handbook for systematic reviews of diagnostic test accuracy of diagnostic tests and markers. The Cochrane Collaboration, London, England.

[68] McAdam AJ. 2000. Discrepant analysis: how can we test a test? J Clin Microbiol 38:2027–2029. doi:10.1128/JCM.38.6.2027-2029.2000 10834948 PMC86720

[69] Linnet K, Kondratovich M. 2004. Partly nonparametric approach for determining the limit of detection. Clin Chem 50:732–740. doi:10.1373/clinchem.2003.029983 14764644

[70] Salanti G. 2012. Indirect and mixed-treatment comparison, network, or multiple-treatments meta-analysis: many names, many benefits, many concerns for the next generation evidence synthesis tool. Res Synth Methods 3:80–97. doi:10.1002/jrsm.1037 26062083

[71] Sutcliffe K, Thomas J, Stokes G, Hinds K, Bangpan M. 2015. Intervention component analysis (ICA): a pragmatic approach for identifying the critical features of complex interventions. Syst Rev 4:140. doi:10.1186/s13643-015-0126-z 26514644 PMC4627414

[72] Thomas J, O’Mara-Eves A, Brunton G. 2014. Using qualitative comparative analysis (QCA) in systematic reviews of complex interventions: a worked example. Syst Rev 3:67. doi:10.1186/2046-4053-3-67 24950727 PMC4079172

[73] Trikalinos TA, Balion CM, Coleman CI, Griffith L, Santaguida PL, Vandermeer B, Fu R. 2012. Chapter 8: meta-analysis of test performance when there is a “gold standard". J Gen Intern Med 27 Suppl 1:S56–S66. doi:10.1007/s11606-012-2029-1 22648676 PMC3364353

[74] Deeks JJ. 2001. Systematic reviews in health care: systematic reviews of evaluations of diagnostic and screening tests. BMJ 323:157–162. doi:10.1136/bmj.323.7305.157 11463691 PMC1120791

[75] Reitsma JB, Moons KG, Bossuyt PM, Linnet K. 2012. Systematic reviews of studies quantifying the accuracy of diagnostic tests and markers. Clin Chem 58:1534–1545. doi:10.1373/clinchem.2012.182568 22991421

[76] Leeflang MM, Deeks JJ, Takwoingi Y, Macaskill P. 2013. Cochrane diagnostic test accuracy reviews. Syst Rev 2:82. doi:10.1186/2046-4053-2-82 24099098 PMC3851548

[77] Bossuyt PM, Reitsma JB, Linnet K, Moons KG. 2012. Beyond diagnostic accuracy: the clinical utility of diagnostic tests. Clin Chem 58:1636–1643. doi:10.1373/clinchem.2012.182576 22730450

[78] Moons KGM, de Groot JAH, Linnet K, Reitsma JB, Bossuyt PMM. 2012. Quantifying the added value of a diagnostic test or marker. Clin Chem 58:1408–1417. doi:10.1373/clinchem.2012.182550 22952348

[79] Rutter CM, Gatsonis CA. 2001. A hierarchical regression approach to meta-analysis of diagnostic test accuracy evaluations. Stat Med 20:2865–2884. doi:10.1002/sim.942 11568945

[80] Higgins JPT. 2003. Measuring inconsistency in meta-analyses. BMJ 327:557–560. doi:10.1136/bmj.327.7414.557 12958120 PMC192859

[81] Rücker G, Schwarzer G, Carpenter JR, Schumacher M. 2008. Undue reliance on I^2^ in assessing heterogeneity may mislead. BMC Med Res Methodol 8:79. doi:10.1186/1471-2288-8-79 19036172 PMC2648991

[82] Freeman SC, Kerby CR, Patel A, Cooper NJ, Quinn T, Sutton AJ. 2019. Development of an interactive web-based tool to conduct and interrogate meta-analysis of diagnostic test accuracy studies: MetaDTA. BMC Med Res Methodol 19:81. doi:10.1186/s12874-019-0724-x 30999861 PMC6471890

[83] Mizutani S, Zhou Y, Tian YS, Takagi T, Ohkubo T, Hattori S. 2023. DTAmetasa: an R shiny application for meta-analysis of diagnostic test accuracy and sensitivity analysis of publication bias. Res Synth Methods 14:916–925. doi:10.1002/jrsm.1666 37640914

[84] Blake DR, Doherty LF. 2006. Effect of perineal cleansing on contamination rate of mid-stream urine culture. J Pediatr Adolesc Gynecol 19:31–34. doi:10.1016/j.jpag.2005.11.003 16472726

[85] Bradbury SM. 1988. Collection of urine specimens in general practice: to clean or not to clean? J R Coll Gen Pract 38:363–365. 3256648 PMC1711498

[86] Holliday G, Strike PW, Masterton RG. 1991. Perineal cleansing and midstream urine specimens in ambulatory women. J Hosp Infect 18:71–75. doi:10.1016/0195-6701(91)90096-q 1679076

[87] Schlager TA, Smith DE, Donowitz LG. 1995. Perineal cleansing does not reduce contamination of urine samples from pregnant adolescents. Pediatr Infect Dis J 14:909–911. 8584324

[88] Schneeberger C, van den Heuvel ER, Erwich JJHM, Stolk RP, Visser CE, Geerlings SE. 2013. Contamination rates of three urine sampling methods to assess bacteriuria in pregnant women. Obstet Gynecol 121:299–305. doi:10.1097/AOG.0b013e31827e8cfe 23344279

[89] Nishimura K, Sugiyama D, Kogata Y, Tsuji G, Nakazawa T, Kawano S, Saigo K, Morinobu A, Koshiba M, Kuntz KM, Kamae I, Kumagai S. 2007. Meta-analysis: diagnostic accuracy of anti-cyclic citrullinated peptide antibody and rheumatoid factor for rheumatoid arthritis. Ann Intern Med 146:797–808. doi:10.7326/0003-4819-146-11-200706050-00008 17548411

[90] Harbord RM, Whiting P. 2009. Metandi: meta-analysis of diagnostic accuracy using hierarchical logistic regression. Strata J 9:211–229. doi:10.1177/1536867X0900900203

[91] Hardy JD, Furnell PM, Brumfitt W. 1976. Comparison of sterile bag, clean catch, and suprapubic aspiration in the diagnosis of urinary tract infection in early childhood. Br J Urol 48:279–283. doi:10.1111/j.1464-410x.1976.tb03021.x 963408

[92] Aronson AS, Gustafson B, Svenningsen NW. 1973. Combined suprapubic aspiration and clean-voided urine examination in infants and children. Acta Paediatr Scand 62:396–400. doi:10.1111/j.1651-2227.1973.tb08126.x 4738132

[93] Pylkkänen J, Vilska J, Koskimies O. 1979. Diagnostic value of symptoms and clean-voided urine specimen in childhood urinary tract infection. Acta Paediatr Scand 68:341–344. doi:10.1111/j.1651-2227.1979.tb05017.x 443034

[94] Ramage IJ, Chapman JP, Hollman AS, Elabassi M, McColl JH, Beattie TJ. 1999. Accuracy of clean-catch urine collection in infancy. J Pediatr 135:765–767. doi:10.1016/s0022-3476(99)70099-5 10586183

[95] Morton RE, Lawande R. 1982. The diagnosis of urinary tract infection: comparison of urine culture from suprapubic aspiration and midstream collection in a children’s out-patient department in Nigeria. Ann Trop Paediatr 2:109–112. doi:10.1080/02724936.1982.11748240 6191624

[96] Deeks JJ, Altman DG. 2004. Diagnostic tests 4: likelihood ratios. BMJ 329:168–169. doi:10.1136/bmj.329.7458.168 15258077 PMC478236

[97] Glas AS, Lijmer JG, Prins MH, Bonsel GJ, Bossuyt PMM. 2003. The diagnostic odds ratio: a single indicator of test performance. J Clin Epidemiol 56:1129–1135. doi:10.1016/S0895-4356(03)00177-X 14615004

[98] Powe CE, Evans MK, Wenger J, Zonderman AB, Berg AH, Nalls M, Tamez H, Zhang D, Bhan I, Karumanchi SA, Powe NR, Thadhani R. 2013. Vitamin D-binding protein and vitamin D status of black Americans and white Americans. N Engl J Med 369:1991–2000. doi:10.1056/NEJMoa1306357 24256378 PMC4030388

[99] Toelle P, Peterli R, Zobel I, Noppen C, Christoffel-Courtin C, Peters T. 2012. Risk factors for secondary hyperparathyroidism after bariatric surgery: a comparison of 4 different operations and of vitamin D-receptor-polymorphism. Exp Clin Endocrinol Diabetes 120:629–634. doi:10.1055/s-0032-1321811 23073920

[100] Trikalinos TA, Balion CM. 2012. Options for summarizing medical test performance in the abscence of a gold standard. J Gen Intern Med 27 Suppl 1:S67–S75. doi:10.1007/s11606-012-2031-7 22648677 PMC3364362

[101] Ioannidis JPA. 2008. Interpretation of tests of heterogeneity and bias in meta-analysis. J Eval Clin Pract 14:951–957. doi:10.1111/j.1365-2753.2008.00986.x 19018930

[102] Ulrich RS, Zimring C, Zhu X, DuBose J, Seo HB, Choi YS, Quan X, Joseph A. 2008. A review of the research literature on evidence-based healthcare design. HERD 1:61–125. doi:10.1177/193758670800100306 21161908

[103] Institute of Medicine. 2009. Roundtable on evidence-based medicine leadership committments to improve value in healthcare: finding common ground workshop summary. National Academic Press, Washington, DC. 21391347

[104] Grol R, Zwaard A, Mokkink H, Dalhuijsen J, Casparie A. 1998. Dissemination of guidelines: which sources do physicians use in order to be informed? Int J Qual Health Care 10:135–140. doi:10.1093/intqhc/10.2.135 9690886

[105] Grimshaw JM, Eccles MP, Lavis JN, Hill SJ, Squires JE. 2012. Knowledge translation of research findings. Implement Sci 7:50. doi:10.1186/1748-5908-7-50 22651257 PMC3462671

[106] Squires JE, Sullivan K, Eccles MP, Worswick J, Grimshaw JM. 2014. Are multifaceted interventions more effective than single-component interventions in changing health-care professionals' behaviours? an overview of systematic reviews. Implement Sci 9:152. doi:10.1186/s13012-014-0152-6 25287951 PMC4194373

[107] Mamdani M, Sykora K, Li P, Normand S-LT, Streiner DL, Austin PC, Rochon PA, Anderson GM. 2005. Reader’s guide to critical appraisal of cohort studies: 2. Assessing potential for confounding. BMJ 330:960–962. doi:10.1136/bmj.330.7497.960 15845982 PMC556348

[108] Masica AL, Richter KM, Convery P, Haydar Z. 2009. Linking joint commission inpatient core measures and national patient safety goals with evidence. Proc (Bayl Univ Med Cent) 22:103–111. doi:10.1080/08998280.2009.11928486 19381308 PMC2666853

[109] Patton KA. 2002. Role of JCAHO standards and clinical practice guidelines in promoting appropriate antimicrobial use. Am J Health Syst Pharm 59:S16–S18. doi:10.1093/ajhp/59.suppl_3.S16 11977862

[110] Zneimer SM, Hongo D. 2021. Preparing for clinical laboratory improvement amendments (CLIA) and college of American pathologists (CAP) inspections. Curr Protoc 1:e324. doi:10.1002/cpz1.324 34958716

[111] Schifman RB, Bachner P, Howanitz PJ. 1996. Blood culture quality improvement: a college of American pathologists Q-probes study involving 909 institutions and 289 572 blood culture sets. Arch Pathol Lab Med 120:999–1002. 12049115

[112] Bekeris LG, Tworek JA, Walsh MK, Valenstein PN. 2005. Trends in blood culture contamination: a college of American pathologists Q-tracks study of 356 institutions. Arch Pathol Lab Med 129:1222–1225. doi:10.5858/2005-129-1222-TIBCCA 16196507

[113] Chawla R, Goswami B, Singh B, Chawla A, Gupta VK, Mallika V. 2010. Evaluating laboratory performance with quality indicators. Lab Med 41:297–300. doi:10.1309/LMS2CBXBA6Y0OWMG

[114] Russell B, Campbell S, Campos JM, Hall GS, LeBar WD, Greene W, Roush D, Rudrik JT, Sautter RL. 2007. The national academy of clinical biochemistry laboratory medicine practice guidelines for point of care infectious disease testing. Point Care 6:231–236. doi:10.1097/POC.0b013e31809f5fc8

[115] Brouwers MC, Kho ME, Browman GP, Burgers JS, Cluzeau F, Feder G, Fervers B, Graham ID, Grimshaw J, Hanna SE, Littlejohns P, Makarski J, Zitzelsberger L, Consortium ANS. 2010. AGREE II: advancing guideline development, reporting, and evaluation in health care. Prev Med 51:421–424. doi:10.1016/j.ypmed.2010.08.005 20728466

[116] Nachamkin I, Kirn TJ, Westblade LF, Humphries R. 2017. Assessing clinical microbiology practice guidelines: American society for microbiology ad hoc committee on evidence-based laboratory medicine practice guidelines assessment. J Clin Microbiol 55:3183–3193. doi:10.1128/JCM.01124-17 28835476 PMC5654901

[117] Don-Wauchope AC, Sievenpiper JL, Hill SA, Iorio A. 2012. Applicability of the AGREE II instrument in evaluating the development process and quality of current national academy of clinical biochemistry guidelines. Clin Chem 58:1426–1437. doi:10.1373/clinchem.2012.185850 22879395

[118] Brouwers MC, Kho ME, Browman GP, Burgers JS, Cluzeau F, Feder G, Fervers B, Graham ID, Grimshaw J, Hanna SE, Littlejohns P, Makarski J, Zitzelsberger L. 2013. User’s manual: instructions for using the AGREE II, appraisal of guidelines for research and evaluation AGREEII instrument. Cnadaian Institute of Health Research, Ottowa, Canada.

[119] Proehl JA, Leviner S, Bradford JY, Storer A, Barnason S, Brim C, Halpern J, Patrick VC, Williams J. 2012. Clinical practice guideline: prevention of blood culture contamination. Emergency Nurses Assocation. Retrieved 28 Aug 2016.

[120] Harrison MB, Légaré F, Graham ID, Fervers B. 2010. Adapting clinical practice guidelines to local context and assessing barriers to their use. CMAJ 182:E78–E84. doi:10.1503/cmaj.081232 19969563 PMC2817341

[121] Shekelle P, Woolf S, Grimshaw JM, Schünemann HJ, Eccles MP. 2012. Developing clinical practice guidelines: reviewing, reporting, and publishing guidelines; updating guidelines; and the emerging issues of enhancing guideline implementability and accounting for comorbid conditions in guideline development. Implement Sci 7:62. doi:10.1186/1748-5908-7-62 22762242 PMC3503794

